# Differential effects of alendronate on chondrocytes, cartilage matrix and subchondral bone structure in surgically induced osteoarthritis in mice

**DOI:** 10.1038/s41598-024-75758-7

**Published:** 2024-10-23

**Authors:** Marianne Ehrnsperger, Shahed Taheri, Patrick Pann, Arndt F. Schilling, Susanne Grässel

**Affiliations:** 1 Clinic of Orthopedic Surgery, Exp. Orthopedics, University of Regensburg, ZMB im Biopark 1, Am Biopark 9, Regensburg, Germany; 2 Department of Trauma Surgery, Orthopedics and Plastic Surgery, University Medicine Göttingen, Göttingen, Germany

**Keywords:** DMM, Osteoarthritis, Bisphosphonate, Alendronate, VDIPEN, ADAMTS-5, Subchondral bone, Cartilage, Micro/nano-CT, Osteoclasts, Chondrocytes, NK1-R, MMP-13, Diseases, Pathogenesis, Rheumatology, Musculoskeletal system, Rheumatic diseases

## Abstract

Bisphosphonates (BP) are considered a treatment option for osteoarthritis (OA) due to reduction of OA-induced microtrauma in the bone marrow, stabilization of subchondral bone (SB) layer and pain reduction. The effects of high-dose alendronate (ALN) treatment on SB and articular cartilage after destabilization of the medial meniscus (DMM) or Sham surgery of male C57Bl/6J mice were analyzed. We performed serum analysis; histology and immunohistochemistry to assess the severity of OA and a possible pain symptomatology. Subsequently, the ratio of bone volume to total volume (BV/TV), epiphyseal trabecular morphology and the bone mineral density (BMD) was analyzed by nanoCT. Serum analysis revealed a reduction of ADAMTS5 level. The histological evaluation displayed no protective effect of ALN-treatment on cartilage erosion. NanoCT-analysis of the medial epiphysis revealed an increase of BV/TV in ALN-treated mice. Only the DMM group had significantly higher SB volume accompanied by decreased subchondral bone surface. Furthermore Nano-CT analysis revealed an increase in trabecular density and number, a decreased BMD and reduced osteophyte formation in the ALN mice. ALN treatment affected bone micro-architecture by reducing osteophytosis with simultaneous increasing subchondral bone plate thickness, trabecular thickness and BMD. Accordingly, ALN cannot be considered as a potential treatment strategy in general, however in a subgroup of patients with high bone turnover in an early-stage of OA, ALN might be an option when applied during a restricted time frame.

**Supplementary Information:**

The online version contains supplementary material available at 10.1038/s41598-024-75758-7.

## Introduction

Osteoarthritis (OA) is a highly prevalent, debilitating disease with a multi-factorial etiology. The main symptom that encourages patients to seek professional help is pain. Despite its high incidence, the underlying molecular mechanisms of OA are still poorly understood. To date, apart from conservative, symptom-oriented treatment methods using physiotherapy, intra-articular drug injections and pain therapy, there is no conclusive causal treatment strategy. Pathophysiological changes in the cartilage, the synovial membrane, the meniscus, the local ligaments, the surrounding muscles, and the subchondral bone (SB) are observed at the joint tissue level during OA pathogenesis. SB has gained major attention in OA research as it is the supportive layer beneath the articular cartilage, therefore providing an important shock absorbing function. Furthermore, SB has hierarchical micropores that provide nutrient supply for the deeper cartilage layers^1,2^. Distinctive alterations have been described for osteoarthritic SB, including sclerosis, changes in the trabecular network, osteophyte formation, development of bone cysts and bone marrow edema, shape changes of the subchondral bone plate (SBP), changes in SBP porosity^3^, and changes in bone mineral density. All together, these changes contribute to altered mechanical properties^2^.

One of the earliest alterations in SB associated with OA are bone marrow lesions (BML) that are thought to develop due to excessive repair responses of micro-damaged regions caused by aberrant mechanical loading^4^. Interestingly, BMLs show a robust correlation with OA-related pain^5^. A possible contributing factor for this phenomenon are highly active osteoclasts that are present in BMLs which can induce sensory innervation of the BMLs and the OA cartilage^6,7^. These osteoclasts are also involved in early OA net bone loss. The increased bone turnover induced by micro-damage repair with increased vascularization leads to growth of SBP pores with enhanced cross-communication of bone and cartilage^8–11^. Clinical hallmarks of late-stage OA include osteophytes at the joint margins (though development may start already early in the disease) and sclerosis of the SB compartment^12,13^. Osteoclasts further may contribute to OA-related early changes in the SB microarchitecture^14,15^. While these effects of osteoclasts play a more pronounced role in early OA, osteoblasts take over in later stages inducing a sclerotic bone phenotype^16^.

In addition to these changes, the increased expression of various nociceptive neuroreceptors and their influence on cartilage cell metabolism are increasingly discussed in the literature. In this context, the direct influence of sensory neuropeptides as substance P (SP) on chondrocyte metabolism has been demonstrated^17,18^. Grimsholm et al. analyzed knee tissues of OA patients where they observed that the expression of NK1-R in cells of the interstitial and synovial tissue in the knee joint may modulate synovial inflammation^19^. Furthermore, the involvement of sensory neurotransmitters in cartilage and bone metabolism was demonstrated recently by our group^20^. The expression of various collagenases, in particular matrix metalloproteinase (MMP) -13, also plays a decisive role in the development of OA. MMP-13 is a product of chondrocytes and degrades not only collagens but also aggrecan creating the neoepitope VDIPEN among others^21^ and thus plays a major role in the destruction of the cartilage matrix^22^.

Several attempts have been made to target SB changes in OA, including the use of bisphosphonates (BPs) (molecules targeting osteoclast activity and differentiation), therapies targeting transforming growth factor β (TGFβ), as well as anabolic strategies using analoga of the parathyroid hormone^23^. So far, these SB-targeting strategies have not matched expectations. Reports about efficacy of BPs in OA are contradictory and a multitude of studies attest limited benefit of BP use in OA pathophysiology. However, a few studies suggest that for specific patient subgroups, the use of BPs might be beneficial^24^. Furthermore, effectiveness might be dependent on the type of BP used for a certain application or patient subgroup, as most BPs display slightly altered effectiveness. In general, BPs such as zoledronic acid and risedronate were most effective in patients diagnosed with BMLs. ALN was not able to effectively relieve pain in early-to-mid-stage knee OA patients with diagnosed BMLs or normalized MRI findings^25^ but could mitigate the pain in hip OA^26^. Conversely, ALN treatment could improve disease progression in various OA animal models. ALN reduced structural deterioration and pain after DMM surgery in mice^7^ whereas its efficacy was ambivalent in OA mice with high- and low-bone masses: in low-bone mass OA mice, ALN treatment exacerbated cartilage degeneration whereas in high-bone mass OA mice, ALN was able to protect from cartilage loss^27^. Moreover, the efficacy of ALN treatment was shown to be dose dependent. High-dose ALN prevented early degenerative changes of bone and cartilage after tibial compressive overload^28^, reduced severe OA progression in a rat model^29^ and ameliorated cartilage and bone changes in a rabbit OA model^30^. However, details on how high-dose ALN influences the musculoskeletal system on a structural and cellular level are not well understood.

In this context, the current study aimed to elucidate how high-dose ALN treatment would affect cartilage and SB changes in a meniscal destabilization (DMM) induced murine OA model.

## Materials and methods

### Animals

Male C57BL/6J mice were purchased from Charles River Laboratories (Sulzfeld, Germany) at the age of 10 weeks and were housed under standard conditions at a 12-hour light/dark cycle. The mice had *ad libitum* access to food and water. All animal experiments were approved by the District Government of Lower Franconia and the national ethics committee (Regierung von Unterfranken, Bavaria, approval code: AZ 55.2-2531-2-289, date of approval July 27th 2016). At the beginning of the experiments, 13 animals at the age of 12 weeks per group and experimental time point were randomly assigned to DMM or Sham surgery and to the respective ALN treatment or non-treatment groups.

All animal experiments were performed in accordance with relevant named guidelines and regulations.

### Surgical induction of osteoarthritis

OA was induced by destabilization of the medial meniscotibial ligament (DMM), a method described by Glasson et al.^31^. Briefly, after intraperitoneal anesthesia with fentanyl, medetomidine and midazolam, a 3 mm skin incision was made between the distal patella and the proximal tibia plateau of the right leg, exposing the knee joint. The joint capsule was opened with a 1–2 mm incision medial to the patellar tendon. For induction of OA, the medial meniscotibial ligament was dissected carefully after visualization using micro scissors. In the control group (Sham), the ligament was only visualized and remained intact. Joint capsule and skin were closed and animals received analgesia (buprenorphine, 0.1 mg/g bodyweight). Immediately after surgery, animals were allowed to move freely and recover from surgery. After 2, 4, 8, and 12 weeks, animals were anesthetized and blood was collected via retro-orbital puncture. Immediately afterwards, the animals were killed under deep anaesthesia by cervical dislocation. Knee joints were prepared and stored at -80 °C until µCT and subsequent histological analysis. For nano-CT analysis, 3 animals per group were killed 8 weeks after surgery and knee joints were prepared and fixed with 4% formalin.

### Alendronate treatment

Starting at the day of DMM and Sham surgery, subgroups of mice received subcutaneous injections of 1 mg/kg body weight ALN (alendronate sodium trihydrate, #A4978, Sigma-Aldrich, St. Louis, Missouri, USA) diluted in 0.9% sodium chloride solution biweekly over the time course of the study. The dose regimen closely adhered to a publication from Khorasani et al.^28^.

### Ultra-high resolution nanoCT analysis

Eight weeks after surgery, knee joints from *N* = 3 mice were prepared and fixed for 16 h in 4% paraformaldehyde and stored in 70% ethanol afterwards. In the first step the knee joints were scanned to get an overview of the mouse knee anatomy, with detailed observations on the femoral condyles, meniscal ossicles, tibial plateau, SB, epiphyseal plate, and the metaphysis. Based on these images, 3D-reconstructed models were generated for visual topographical inspection of the mouse knee. In the following nano-CT-analysis (Scanco Medical, Brüttisellen, Switzerland; DFG number: 3230/30009760) was used for quantitative evaluation of subchondral bone plate (SBP) thickness, calcified cartilage (CC), bone volume to total volume (BV/TV), bone mineral density (BMD), trabecular thickness (Tb.Th.) and trabecular number (Tb.N.) and was acquired with the following parameters: 2.0 μm voxel size, 90 kVp, 88 µA and 1500 ms. Nano-CT has a high spatial resolution and integration time. The applied threshold settings, and reorientation of the images were performed as described^32^. The epiphyseal trabecular morphometry was measured in a volume of interest (VOI) of approx. 0.25 mm^3^ between the medial SBP and the epiphyseal plate, while excluding the SBP, cortical bone, as well as the epiphyseal plates from the segmentation. The subarticular trabecular bone was measured starting at 500 μm below the epiphyseal plate for a VOI of approx. 1.5 mm^3^. To measure the volume and the mineral density of the osteophytes, they were manually segmented from the medial tibial condyle, taking the intersection line between the two features as the segmentation landmark. The length of the medial condyle (as an indicator for osteophyte formation) as well as the lateral condyle were measured at 300 μm distally from the most superficial surface of the SB. The length was defined as the distance from the center of the condyle in proximity of the trochlear groove to the medial prominence. The anterior meniscal ossicles were contoured manually and segmented using the OpenVMS software (Scanco Medical, Brüttisellen, Switzerland). The VOI was set to approx. 0.5 mm^3^ for the Sham-operated mice and approx. 1.3 mm^3^ for the DMM-operated mice owing to the irregular surface expansion of the ossicles along the long axis of the tibia. To calculate the medial SBP thickness, the SBP and the CC were roughly segmented with respective lower threshold values of 850 mg HA/cm^3^ and 520 mg HA/cm^3^. The medial SBP thickness was then measured in three equally-spaced coronal sections of each knee joint, in at least 40 fixed spots, excluding the intercondylar eminence. However, as the greyscale values of these two adjacent layers are in the same proximity, the segmentation can be improved by an interactive step based on a modified Seeded Region Growing technique^33^. This semi-automatic step was implemented to extract the CC layer from the underlying subchondral bone. The thickness of the CC layer was then measured directly in 3D with the OpenVMS software (Scanco Medical, Brüttisellen, Switzerland). Color maps of the thickness were obtained from the bins of values and subsequent histograms. To enhance the visualization, the maximum value of the color legends was scaled to 80 μm for all samples.

### Histology and OARSI-score

After nanoCT scanning, samples were fixed in 4% paraformaldehyde for 16 h and decalcified in 20% EDTA for 5 weeks. Paraffin-embedded samples were cut into 6 μm frontal sections using a microtome (Leica Microsystems Nussloch GmbH, Germany). Cartilage degeneration was analyzed in 5–6 sections in 60–90 μm intervals through the weight bearing knee region. For this purpose, sections were deparaffinized, rehydrated and stained with Safranin O, Weigert’s iron hematoxylin, and Fast Green. The stained sections were then scored by two blinded, independent observers according to the OARSI guidelines with little modifications (Table 1)^34^. Sections were scanned with 10x magnification using the TissueFAXS system (DFG code: INST 89/341-1 FUGG) from TissueGnostics (Vienna, Austria). Maximum OARSI scores of the medial femur condyle and the medial tibia plateau were combined, averaged and displayed in the graphs.

Furthermore, medial tibial osteophyte size (0 = none, 1 = small, approx. same thickness as the adjacent cartilage, 2 = medium, 1–3× the thickness of the adjacent cartilage, 3 = large,>3× the thickness of the adjacent cartilage) and maturity (0 = none, 1 = predominantly cartilaginous, 2 = mix of cartilage and bone, 3 = predominantly bone) were evaluated in the same sections used for the OARSI score according to the grading system developed by C. Little^35^. Size and maturity scores were each averaged over the number of sections analyzed per animal and recorded. In addition, the sum of both scores is presented.

**Table 1:** OARSI guidelines for assessment of murine articular cartilage degradation. (modified from Glasson et al.^34^).

Grade	Osteoarthritic damage
0	Normal
0.5	Loss of Safranin O staining without structural changes
1	Small fibrillations without loss of cartilage/uneven surface
2	Vertical clefts and erosions down to the layer immediately below the superficial layer and some loss of surface lamina
3	Vertical clefts/erosions to the calcified cartilage < 25% of the articular surface
4	Vertical clefts/erosions to the calcified cartilage 25–50% of the articular surface
5	Vertical clefts/erosions to the calcified cartilage 50–75% of the articular surface
6	Vertical clefts/erosions to the calcified cartilage > 75% of the articular surface

### Osteoclast staining and quantification

Sections were deparaffinized, rehydrated and stained for tartrate-resistant acid phosphatase (TRAP) using the “Acid phosphatase, leukocyte (TRAP) Kit” from Sigma (ST. Louis, MO, USA). Sections were scanned at 20x magnification using the TissueFAXS system (DFG code: INST 89/341-1 FUGG) from TissueGnostics (Vienna, Austria). Adobe Photoshop CS4 (San Jose, CA, USA) was used to manually count osteoclasts in the four compartments of the SB (medial femur condyle/tibia plateau, lateral femur condyle/tibia plateau) and to determine the length of the respective bone surface. Three sections through the weight bearing region and one section in the non-bearing region (proximal to the patellar region) were analyzed. Only cells with a red-brown staining directly attached to the bone surface were counted as osteoclasts.

### Immunohistochemical semi-quantification of the NK1-R, aggrecan neoepitope VDIPEN, COMP, collagen II, RUNX2 and MMP-13 in chondrocytes

Paraffin-embedded samples were cut into 6 μm frontal sections using a microtome (Leica Microsystems Nussloch GmbH, Germany) as described above. Expression of the NK1-R was analyzed on 8 sections, aggrecan-neoepitope VDIPEN, collagen II and COMP on 2 to 5 sections, RUNX2 on 2 sections and MMP-13 on 1 section per animal. For this purpose, the sections were deparaffinized and rehydrated. Unmasking for the staining of NK1-R, collagen II and the aggrecan neoepitope VDIPEN was performed enzymatically with 0.05% protease XXIV (0.05% in PBS; Sigma) for 10 min at 37 °C, followed by 0.1% hyaluronidase for 90 min at 37 °C. Unmasking of COMP was performed with hyaluronidase (10 mg/ml in PBS pH5.5) for 15 min at room temperature in a humid chamber. For visualizing MMP-13 and RUNX2, heat demasking was performed with citrate buffer at 60 °C for 24 h. 3% H_2_O_2_ was applied for 10 min to suppress endogenous peroxidase activity, COMP was blocked with 3% H_2_O_2_ at room temperature for 5 min to suppress endogenous peroxidase activity. For NK1-R, RUNX2 and MMP-13, blocking was performed with 0.5% goat serum for 60 min at room temperature; VDIPEN and collagen II were blocked with 1% bovine serum albumin and 5% normal goat serum for 60 min in a humid chamber at room temperature. COMP was blocked with 2.5% horse serum and 5% normal goat serum for 60 min at room temperature in a humid chamber. This was followed by overnight incubation with the primary antibody at 4 °C. The following dilutions (Signal Stain Antibody Diluent, Cell Signaling #8112) were used for the primary antibodies: for NK1R 1:400 (Abcam Rabbit monoclonal; ab 183713), for MMP-13 1:500 (Abcam Rabbit polyclonal; ab 39012), for RUNX2 1:260 (Abcam Rabbit monoclonal; ab 192256) for VDIPEN 1: 2500 (rabbit polyclonal; GP1-PEN)^36^ in blocking solution, for collagen II (abcam rabbit polyclonal; ab 34712) 1:400 in blocking solution and for COMP 1:500^37^ in 1% bovine serum albumin in TBS. The isotype control was performed for the NK1R antibody with a non-specific monoclonal rabbit antibody at a dilution of 1:728 (abcam rabbit monoclonal, ab 172730), for RUNX2 with a mouse IgG2a diluted 1:888 (Abcam, ab18414) and for MMP-13 with rabbit IgG antibody (Novus Bio, NBP1-97014) at a dilution of 1: 500. As isotype control for VDIPEN and COMP we used a polyclonal rabbit antibody at a dilution of 1:500 (abcam rabbit polyclonal, ab 27478), and for Collagen II a polyclonal rabbit antibody at 1:800 (abcam rabbit polyclonal, ab 27478). The following day, the secondary antibody coupled with HRP (Signal Stain Boost IHC Detection Reagent; Cell Signaling #8114) was applied. Visualization was carried out using DAB substrate (3,3’-diaminobenzidine Liquid Substrate System tetrahydrochloride; Sigma #D7304). Counterstaining was performed with Gill-III-hematoxilin (Merck 1.05174.0500). NK1R-, MMP-13- and RUNX2-sections were scanned with 20x magnification using the TissueFAXS system (DFG code: INST 89/341-1 FUGG) from TissueGnostics (Vienna, Austria). The sections of VDIPEN, COMP and Collagen II were scanned with 10x magnification using the Keyence BZ-X810 microscope (Keyence Deutschland GmbH, Neu-Isenburg). Adobe Photoshop CS4 (San Jose, CA, USA) was used to manually count chondrocytes in the four articular cartilage knee compartments (medial and lateral femur condyle and tibia plateau). The region to be counted was first delimited manually, with subsequent calculation of the number of pixels of the area to be counted. This was followed by manual counting with the Adobe Shop counting tool; the counted number of positive stained chondrocytes was then related to the total number of cells and to the calculated area as percentage.

### Serum analysis

Blood was collected and serum was prepared after centrifugation. Murine tartrate-resistant acid phosphatase form 5b (TRAcP5b, MouseTRAP™, SB-TR103, IDS Immunodiagnostic Systems, Frankfurt/Main, Germany) and carboxy-terminal telopeptide of type I collagen (CTX-I, CEA665Mu, Cloud-Clone Corp., Houston, TX, USA) were analyzed as markers for osteoclast activity and bone degradation respectively, according to the manufacturer instructions. Serum interleukin-1β (IL-1β) concentrations were analyzed using the Mouse IL-1 beta/ IL-1F2 DuoSet ELISA (DY401-05) in conjunction with the DuoSet Ancillary Reagent Kit 2 (DY008, both R&D systems by Bio-techne, Minneapolis, MN, USA). ELISA Kits for the detection of osteoprotegerin (OPG) and dickkopf-1 (Dkk-1) were purchased from Boster Bio (OPG #EK0481, Dkk-1 #EK0925, Pleasanton, CA, USA) and used according to the manufacturer’s instructions. Serum ADAMTS5 concentrations were analyzed using the Mouse ADAMTS5 ELISA Kit (MBS7606113, MyBioSource.com, San Diego, CA, USA).

### Statistical analysis

GraphPad prism 6 (San Diego, CA, USA) was used to prepare all graphs and to perform statistical analysis. One-way analysis of variance (ANOVA) and Bonferroni post-hoc test or Mann-Whitney-Test were applied to test for significant differences between the groups at different time points. The t-test was used for the statistical analysis of immunohistological data of NK1-R, MMP-13, VDIPEN and RUNX2, for the statistical analysis of COMP we use One-Way-ANOVA followed by Kruskal-Wallis-test.

No statistical analysis was possible for the evaluation of collagen II; an optical comparison of the color intensity of the cartilage matrix was performed.

## Results

### Serum analysis of marker molecules related to bone and cartilage remodeling

**Fig. 1:**
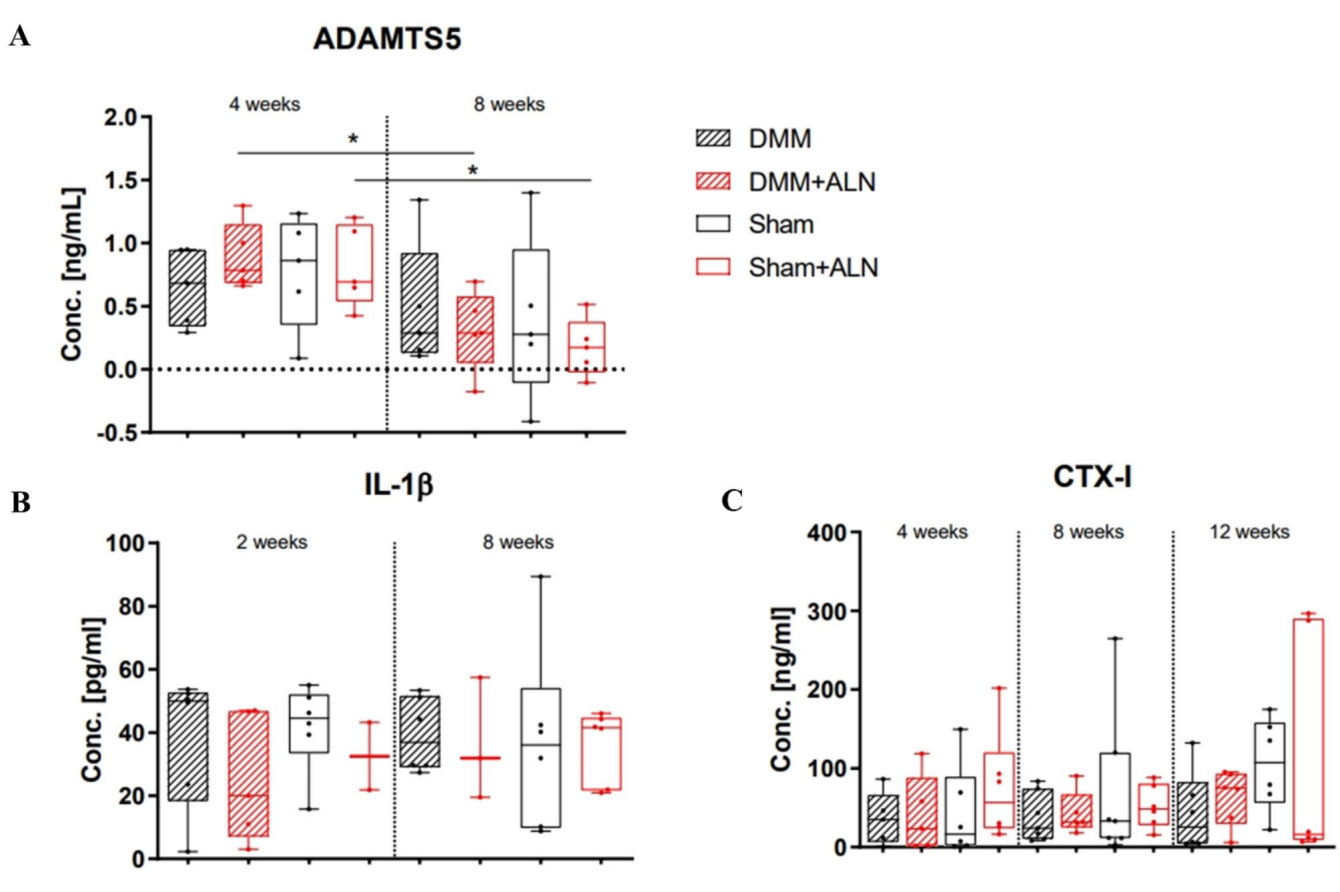
Analysis of ADAMTS5, IL-1b and CTX-I serum concentrations in untreated and ALN-treated mice after DMM or Sham surgery. (**A**) ADAMTS5 concentration was determined in the serum in untreated and ALN-treated mice 4 and 8 weeks after DMM or Sham surgery. Mann-Whitney-Test, *p < 0.05, **p < 0.01. *N* = 5. (**B**) IL-1ß serum concentration was determined in the serum in untreated and ALN-treated mice 2 and 8 weeks after DMM or Sham surgery. One-way ANOVA, *N* = 2–6. (**C**) Bone turnover marker CTX-I was determined in the serum of untreated and ALN-treated mice 4, 8 and 12 weeks after DMM or Sham surgery. One-way ANOVA, *N* = 5–7.

To address in vivo effects of ALN on OA progression, we studied a panel of molecules related to modulation of bone resorption, cartilage degeneration and osteoclast- and osteoblast activity in the serum of untreated (controls) and ALN-treated DMM/Sham mice. The major aggrecanase degrading articular cartilage, ADAMTS5, was found to be significantly reduced in the ALN groups after 8 weeks in comparison to 4 weeks, both after Sham and DMM surgery (Fig. 1A). However, we did not detect differences in ADAMTS5 serum concentration between the ALN treatment groups and the untreated groups at both analysis time points and neither between DMM and Sham groups. Analysis of serum IL-1β concentrations of untreated and ALN-treated mice 2 and 8 weeks after DMM or Sham surgery did not reveal altered serum levels (Fig. 1B). Likewise, serum concentrations of CTX-I, the main collagen I degradation product related to bone degradation, remained mainly unaffected throughout the experimental time course (Fig. 1C). Additional analysis at 12 weeks revealed little impact of OA induction or ALN treatment on the expression of the osteoclast activity marker TRAcP5b (Suppl. Figure S1A), the Wnt-pathway inhibitor DKK-1 (Suppl. Figure S1B) or the decoy receptor for RANKL, osteoprotegerin (OPG, Suppl. Figure S1C).

### Cartilage degradation after OA induction and ALN treatment

**Fig. 2:**
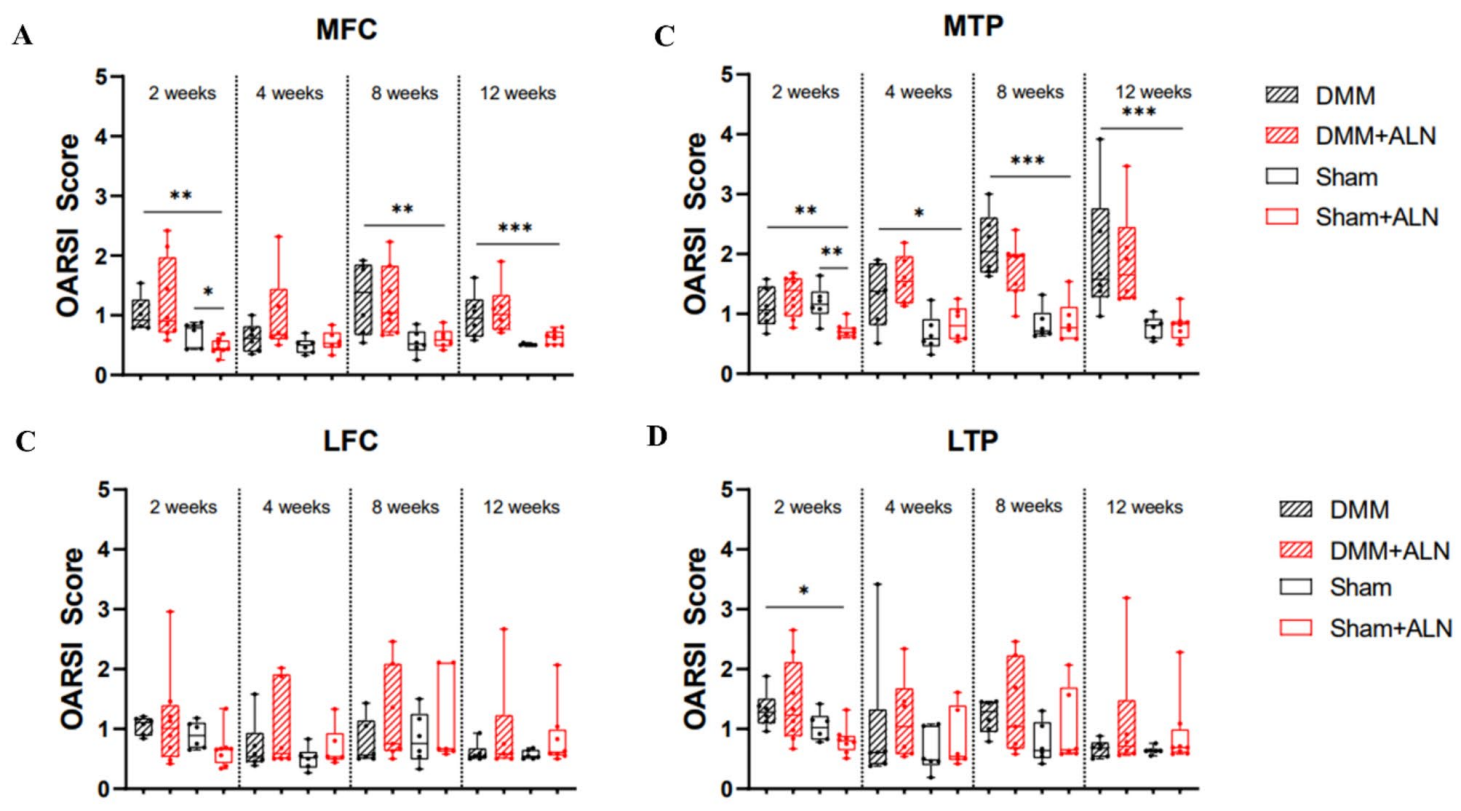
Analysis of cartilage degradation in untreated and ALN-treated mice after DMM and Sham surgery. (**A**-**D**) OARSI scores of the medial and lateral tibia and femur of untreated and ALN-treated mice 2, 4, 8 and 12 weeks after DMM or Sham surgery. Maximum scores of the tibia and femur were averaged. One-way ANOVA followed by Bonferroni post-hoc test. *p < 0.05, **p < 0.01, ***p < 0.001, ****p < 0.0001. *N* = 6. Box plots show median and whiskers from min to max. MFC = medial femoral condyle; MTP = medial tibia plateau; LFC = lateral femoral condyle; LTP = lateral tibia plateau.

**Fig. 3:**
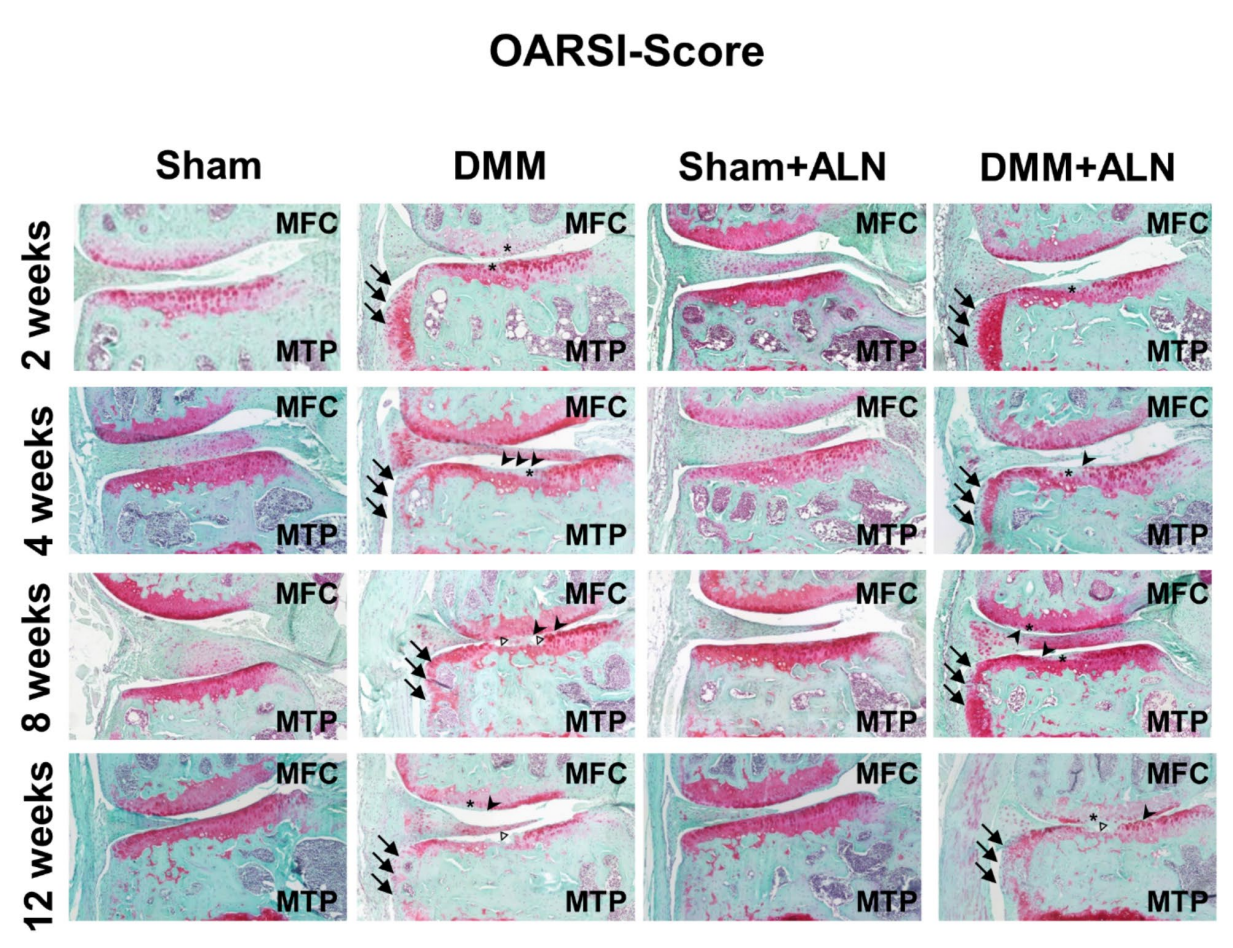
Representative knee images used for OARSI-scores. Representative images of Safranin-O stained frontal sections of paraffin embedded knee joints from untreated and ALN-treated mice 2, 4, 8 and 12 weeks after DMM or Sham surgery. Cartilage of the medial tibia plateau (MTP), medial femoral condyle (MFC), lateral tibia plateau (LTP) and lateral femoral condyle (LFC) was evaluated for grades of cartilage destruction according to the OARSI guidelines. Asterix (*) indicates discoloration due to proteoglycan loss; full arrows indicate osteophyte formation; arrow heads indicate irregular cartilage surface and open triangles indicate cartilage erosions down to the calcified cartilage zone.

Even though ALN treatment mainly targets bone tissue, interference with bone homeostasis at the osteochondral unit might affect cartilage degradative processes induced by the DMM surgery. We therefore looked more closely at ALN effects directly at the articular cartilage level. Cartilage degradation was increased in the medial knee compartments of the DMM mice compared to the corresponding Sham groups, which was reflected in significantly increased OARSI scores in the MFC and MTP (Fig. 2A, B). This effect was absent in the lateral compartments (Fig. 2C, D). It is noteworthy that by trend ALN treatment led to a general reduction in OARSI scores in the Sham groups after 2 weeks with a significant reduction in the MTP of the Sham group. ALN treatment had no effect on cartilage degradation in the DMM groups (Fig. 2A-D). Representative Safranin-O-stained knee sections for each group and time-point are shown in Fig. 3.

Located directly adjacent to the articular cartilage is the calcified cartilage (CC) layer, the transitional region between cartilage and SB. The mean thickness of the medial tibial CC was significantly increased by OA induction after 8 weeks compared to the Sham mice in both treated and untreated groups, while no ALN-specific effect was observed (Suppl. Figure 2A and B).

**Fig. 4:**
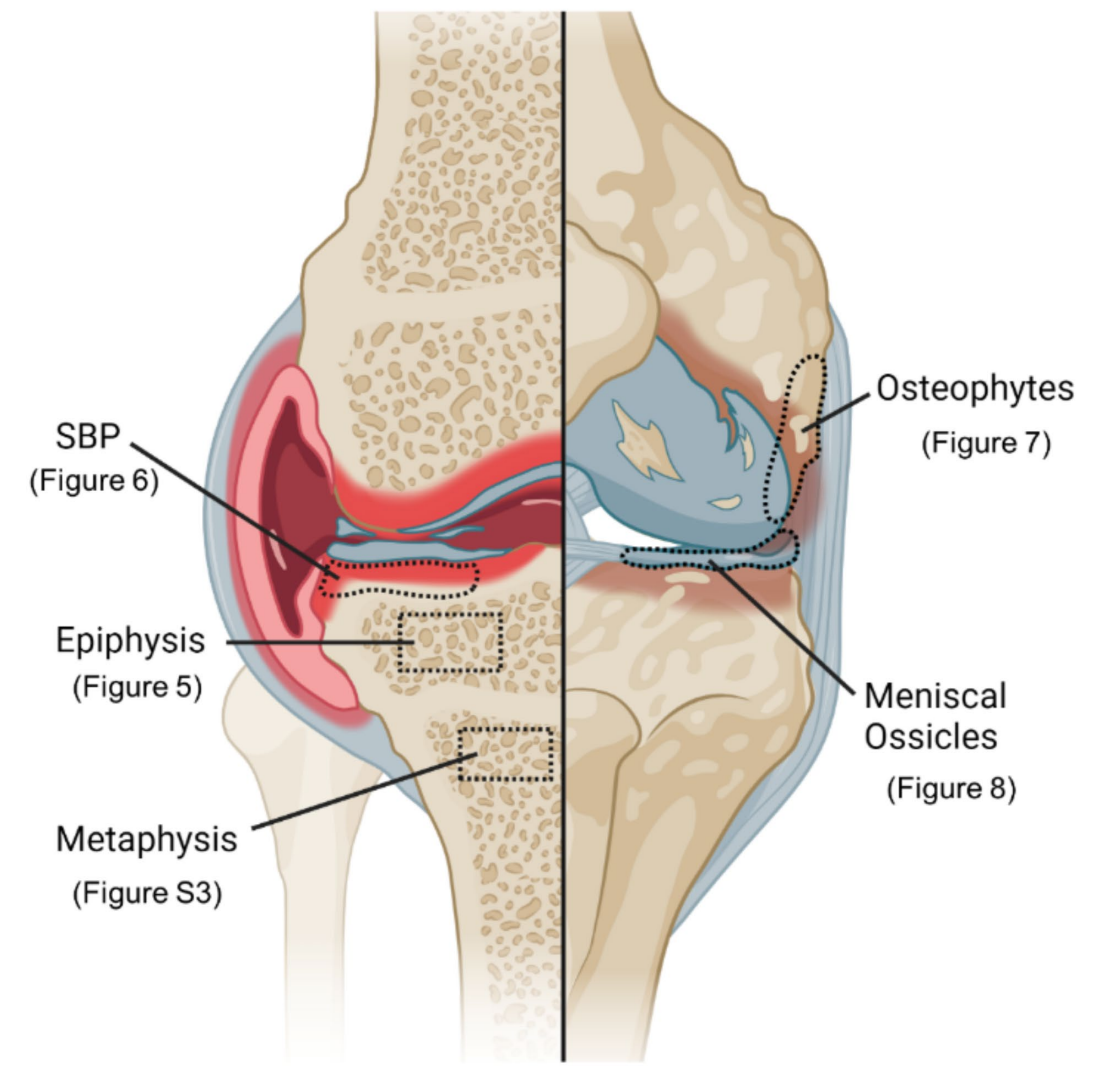
This schematic overview highlights the different ROIs chosen for nanoCT analysis from the different bone regions. Text in brackets indicates the representative figure.

### ALN treatment altered subchondral bone structure of the medial tibia

BPs classically target and inhibit osteoclast activity and are therefore most effective in conditions with high bone turnover. In human OA, predominantly early stages are associated with an increase in the bone turnover rate.

**Fig. 5:**
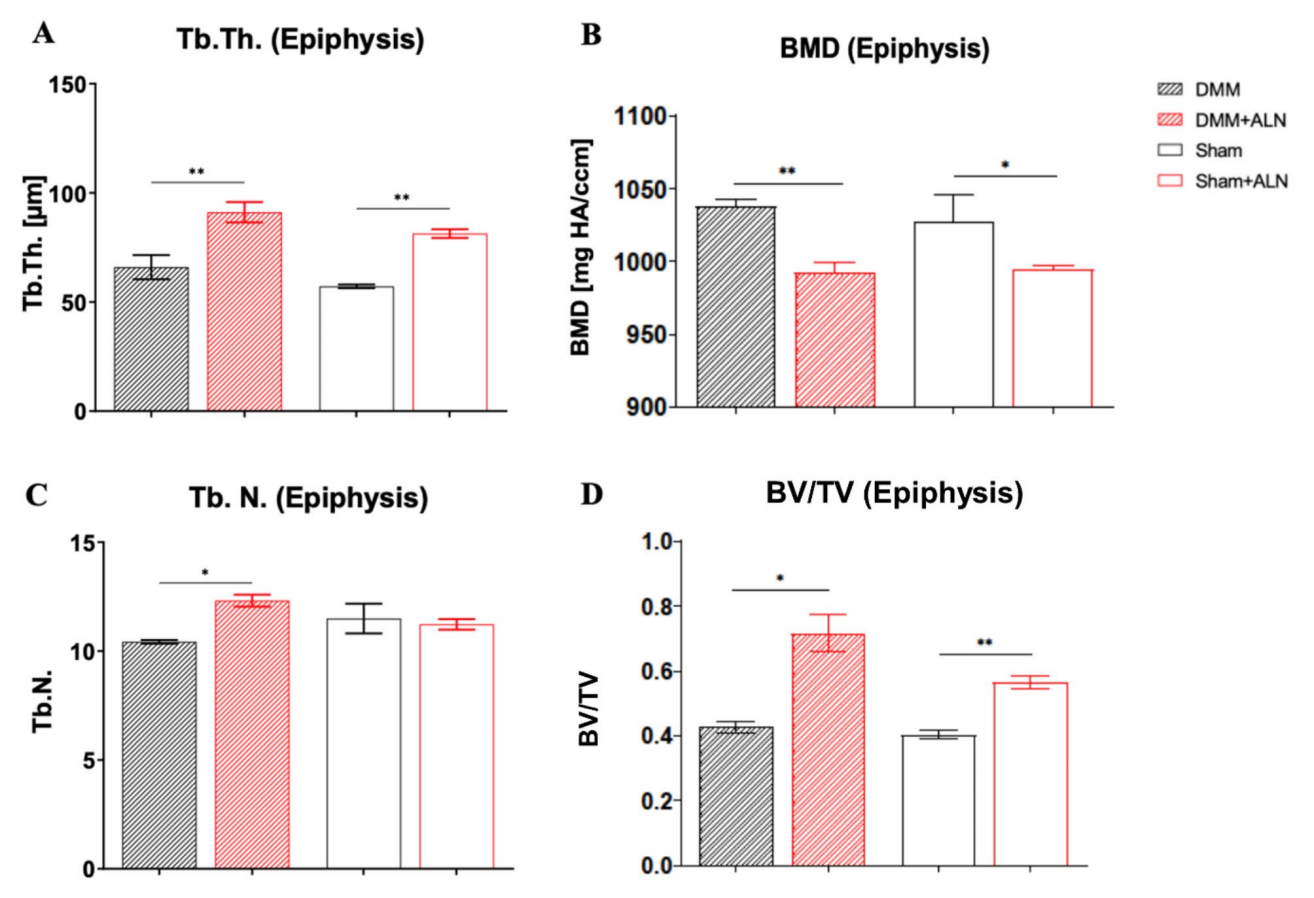
Effect of ALN treatment on epiphyseal bone parameters 8 weeks after DMM and Sham surgery. (**A**-**D**) Analysis represents the trabecular thickness (Tb.Th.; **A**) bone mineral density (BMD; **B**), trabecular number (Tb.N.; **C**) and the total bone volume fraction (BV/TV, **D**) in the epiphyseal region. Bars with median and 95% confidence interval. One-way ANOVA followed by Bonferroni post-hoc test. *p < 0.05, **p < 0.01,***p < 0.001. *N* = 3.

8 weeks after the DMM and Sham surgery nano-CT scans enabled a more detailed analysis of the epiphyseal tibial bone. ALN treatment induced higher epiphyseal Tb.Th. as well as a lower BMD in the epiphysis irrespective of the surgery type compared to the untreated mice (Fig. 4A, B). Though highly significant, BMD reduction by ALN treatment was moderate with a 4.4% reduction in DMM mice and a 3.2% reduction in the Sham groups. Epiphyseal Tb.N. significantly increased in ALN-treated DMM mice compared to the untreated DMM mice (Fig. 5C). BV/TV of the medial tibial SB increased significantly in ALN-treated mice compared to the untreated mice (Fig. 5D). Also the total bone volume fraction increased under ALN-treatment (Fig. 5D).

Additionally, SBP thickness of the medial tibia plateau of both ALN-treated mice groups (DMM and Sham) was significantly increased compared to their respective untreated groups 8 weeks after DMM or Sham surgery (Fig. 4A). Furthermore, the medial SBP thickness of ALN-treated mice was significantly higher compared to the respective lateral side of the same knee; an effect that was not observed in the untreated mice.

**Fig. 6:**
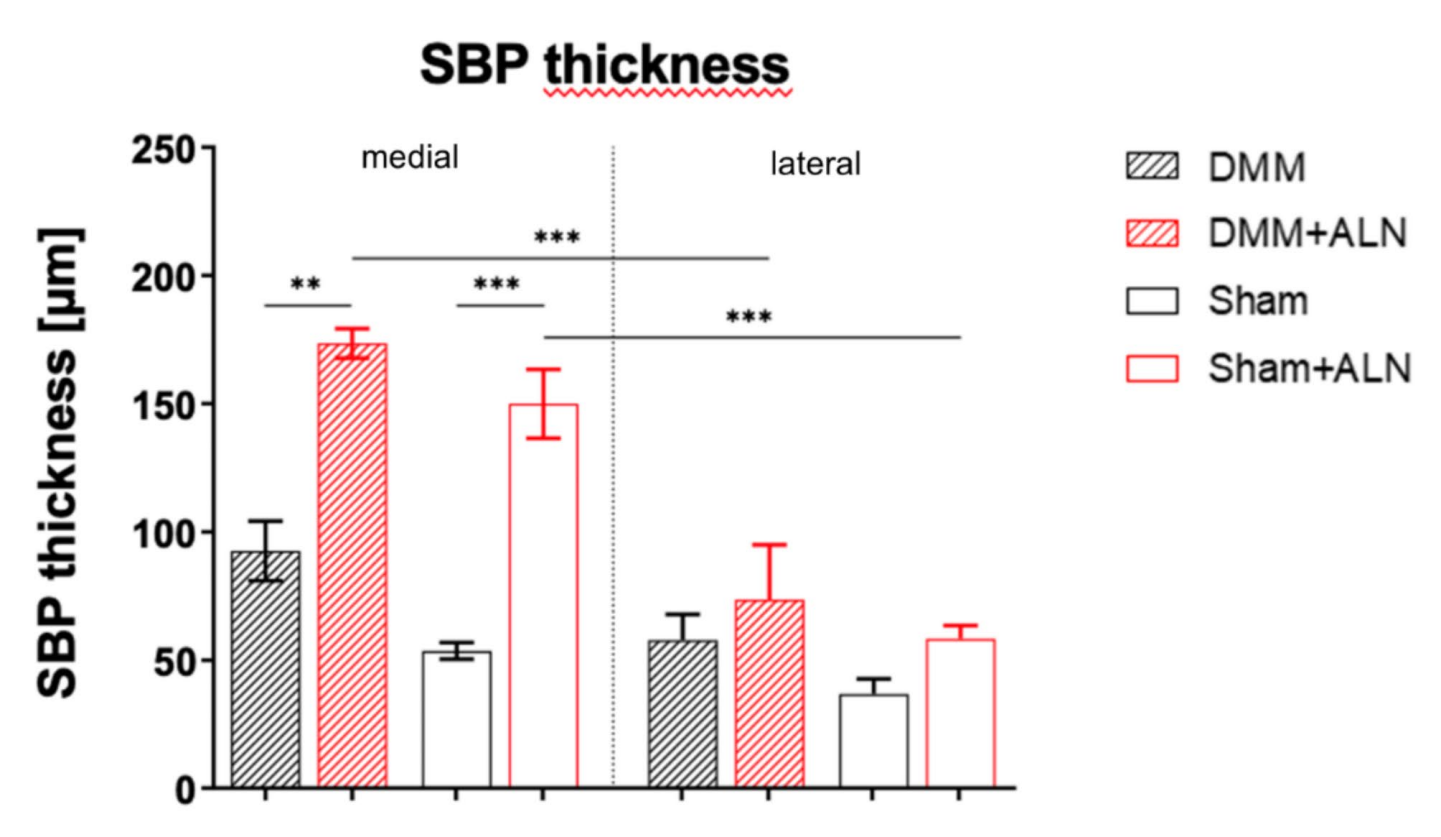
Effect of ALN treatment on SBP thickness 8 weeks after DMM and Sham surgery. (**A**) 8 weeks after surgery in depth analysis of the epiphyseal bone of untreated and ALN-treated mice after DMM or Sham surgery was performed using nano-CT analysis resolving the subchondral bone plate (SBP) thickness of the medial and lateral tibial plateau. Bars with median and 95% confidence interval. One-way ANOVA followed by Bonferroni post-hoc test. *p < 0.05, **p < 0.01,***p < 0.001. *N* = 3.

Apart from the epiphysis, we also analyzed the metaphyseal trabecular (i.e. subarticular) bone compartment adjacent to the growth plate. In this region, BV/TV and Tb.Th. were increased by ALN treatment but remained unaffected by OA induction (Suppl. Figure 3A, B and Fig. 4). No effects of ALN treatment or OA induction were observed regarding the metaphyseal BMD and Tb.N. (Suppl. Figure 3C, D).

### ALN altered patterns of osteophyte and meniscal ossicle formation

A hallmark of OA is the formation of new bone at the joint margins, the osteophytes, appearing as an irregular bony protrusion at the medial tibia plateau (Fig. 4A) as well as the increased surface expansion of meniscal ossicles in the DMM mouse model (Fig. 8E).

**Fig. 7:**
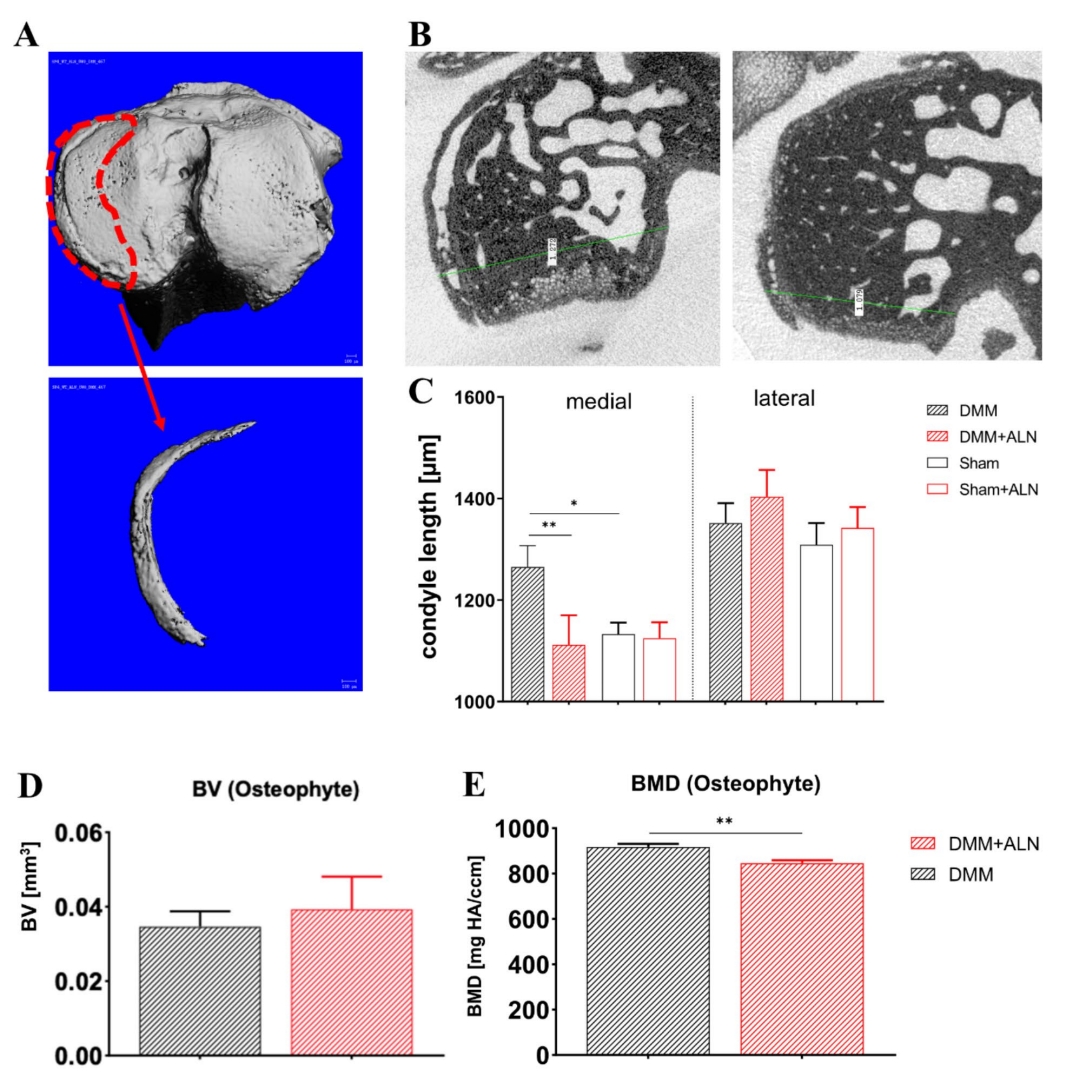
Analysis of osteophyte formation after ALN treatment and DMM and Sham surgery. (**A**) Representative images of the femoral condyle for determination of osteophytosis (red circled area) after induction of OA. (**B**) Tibia plateau length was measured as a mean to identify osteophyte formation at the medial and lateral tibial plateau in untreated and ALN-treated mice 8 weeks after DMM or Sham surgery. (**C**) Quantitative assessment of medial and lateral condyle length in untreated and ALN treated DMM and Sham mice. *N* = 3. (**D**, **E**) Evaluation of osteophyte parameters in untreated DMM and ALN-treated DMM mice 8 weeks after OA induction using nanoCT. Determination of osteophyte bone volume (BV; **D**) and bone mineral density (BMD; **E**). *N* = 3. Bars with median and 95% confidence interval. One-way ANOVA followed by Bonferroni post-hoc test. *p < 0.05, **p < 0.01,***p < 0.001, ****p < 0.0001. *N* = 3.

In the DMM model, it was observed earlier that osteophytes can develop as early as 2 weeks after the surgery^32^. All mice from DMM groups exhibited new bone formation at the medial margins of the knee joint by growing osteophytes over time. The osteophyte formed as an irregular structure at the medial subchondral bone margin, which was accompanied by an increase of the diameter of the medial tibial plateau. The length of the medial and lateral tibia plateaus were determined as indicators of osteophyte formation and structural adjustment to tibia plateau, respectively. DMM surgery induced a significant lengthening of the medial tibia plateau after 8 weeks in the untreated mice compared to the ALN treated group indicating a reduced osteophytosis after ALN treatment (Fig. 7B, C). A closer look at the osteophyte formation after DMM surgery suggested that in ALN-treated mice, the osteophyte mineralization was reduced compared to the untreated animals. While the BV of osteophytes did not differ between the untreated and ALN-treated DMM mice (Fig. 7D), BMD was significantly decreased about 7.7% in the ALN group (Fig. 7E). Additionally, we used an osteophyte size score to grade Safranin O-stained paraffin sections (Suppl. Figure 4A). Osteophytes of ALN treated DMM mice had a smaller size by trend at 8 weeks but a larger size by trend at 12 weeks compared to the untreated DMM mice. To refine the histological osteophyte analysis, a maturity score was applied categorizing osteophytes with high proteoglycan content (score 1) up to almost complete bony structure (score 3). Osteophytes from ALN treated DMM mice at 4 and 8 weeks after surgery were graded less mature by trend than osteophytes from untreated DMM mice indicating a higher bone portion whereas at 12 weeks the osteophytes were graded more mature by trend (Suppl. Figure 4B). The summed score for osteophyte size and maturity confirms this observation (Suppl. Figure 4 C).

Furthermore, calcified cartilage was separated from the denser SBP by a modified seeded region growing approach and computed into colored heat maps (green < < red in mineralization, Suppl. Figure 2). DMM groups showed a consistently higher CC thickness compared to their sham counterparts, with no effect of ALN-treatment (Suppl. Figure 2A).

**Fig. 8:**
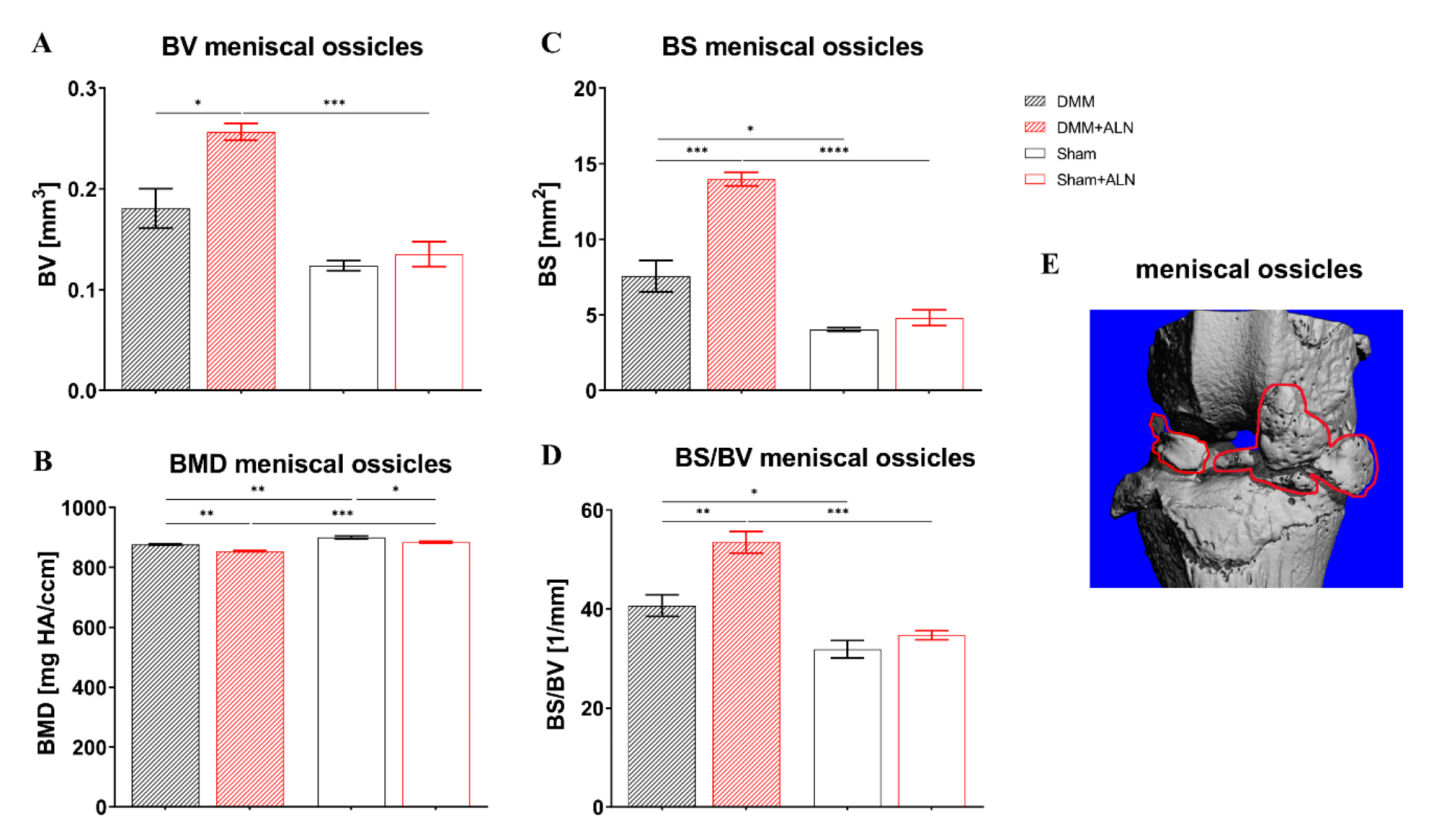
Analysis of meniscal ossicle parameters after ALN treatment and DMM and Sham surgery. (**A**-**D**) By nanoCT analysis, bone volume (BV; **A**), bone mineral density (BMD; **B**), bone surface (BS; **C**) and bone surface density (BS/BV; **D**) of meniscal ossicles were analyzed in untreated and ALN-treated mice 8 weeks after DMM or Sham surgery. Bars with median and 95% confidence interval. One-way ANOVA followed by Bonferroni post-hoc test. *p < 0.05, **p < 0.01,***p < 0.001, ****p < 0.0001. *N* = 3. (**E**) Representative image of the meniscal ossicle formation (red outline) after induction of OA.

High-resolution analysis of meniscal ossicles revealed further effects of the ALN treatment. BV of the meniscal ossicles was increased by DMM surgery (8 weeks) in ALN treated mice compared to untreated DMM mice and the untreated Sham group while no difference in BV was observed between untreated DMM/Sham groups (Fig. 4 A). In general, OA induction (untreated mice 2.6%; ALN-treated mice 3.4%) and ALN treatment (2.6% in DMM and 1.8% in Sham groups) impaired the mineralization leading to a reduced BMD of the meniscal ossicles (Fig. 8B). Bone surface (BS) and bone surface to bone volume ratio (BS/BV) were both increased by OA induction independent of the ALN treatment, but the effect was stronger in ALN-treated DMM mice compared to untreated DMM mice (Fig. 8C, D).

### Osteoclast numbers in the subchondral tibia were not affected by ALN

In vivo, ALN treatment should predominantly inhibit osteoclasts activity and therefore halt bone resorption in areas of high bone turnover which has been described for OA subchondral bone^10^.

**Fig. 9:**
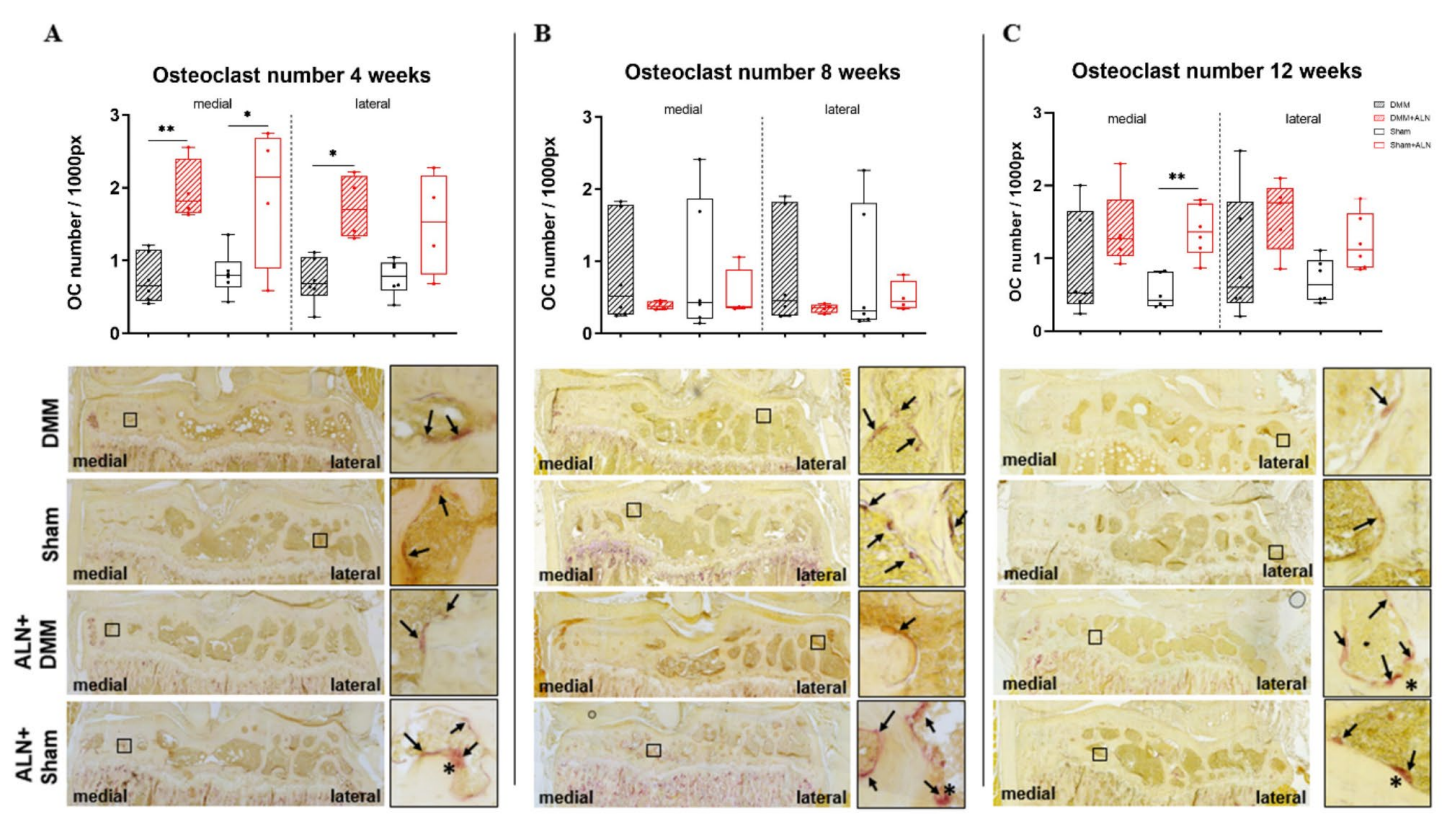
Influence of OA induction and ALN treatment on subchondral osteoclast numbers. (**A**-**C**) Mean osteoclast (OC) numbers per bone surface (in pixel, px) were determined from the medial and lateral femoral and tibial subchondral bone. Comparison of OC/px of untreated and ALN-treated mice at 4 (**A**), 8 (**B**) and 12 (**C**) weeks after DMM and Sham surgery. Box plots show median and whiskers from min to max. One-way ANOVA followed by Bonferroni post-hoc test. *p < 0.05, **p < 0.01. *N* = 4–6.

Analysis of subchondral osteoclasts revealed a larger number in the SB of the medial and lateral tibia of DMM and Sham animals 4 weeks after ALN treatment (Fig. 9A). 8 weeks after surgery, the difference in osteoclast numbers between the untreated and the ALN-treated groups were not significant anymore (Fig. 9B). With progression to late-stage OA (12 weeks), osteoclast numbers increased in the medial compartment of the ALN treated Sham mice while in all other groups, ALN- treatment increased osteoclast number only by trend. (Fig. 9C). Additionally, the representative TRAP-staining images presented in Fig. 9A-C indicate that ALN induced the formation of very large osteoclasts (details in the appropriate boxed images, indicated by arrows) in the SB area. With progression of OA and mouse age, the bone surface area of the medial SB seems to decrease due to sclerotic alterations, but the number of osteoclasts per surface length (per 1000 pixels) remained relatively stable over the time course of 12 weeks.

Images underneath each graph are representative for TRAP-stained frontal knee joint sections from untreated and ALN-treated mice at 4 (A), 8 (B) and 12 (C) weeks after DMM or Sham surgery. Enlargements from the overview pictures (boxed area) show osteoclasts (red staining, black arrows) at the bone surface. ALN treatment induced the formation of large osteoclasts (asterisk).

### ALN treatment effects on the total chondrocyte count

When calculating the total chondrocyte count per area, ALN therapy had no effect in the MFC of DMM and Sham mice after 4 and 8 weeks (Suppl. Figure S5A). In the MTP, the cell count was significantly reduced in ALN treated Sham mice after 4 weeks (Suppl. Figure S5B). By trend, ALN treatment resulted in a moderate increase in the total cell count at the LFC after 4 weeks (Suppl. Figure S5C). After 8 weeks a significant increase in the chondrocyte count was observed in the LTP of ALN treated Sham and DMM mice (Suppl. Figure S5D).

### ALN reduced NK1-R positive chondrocytes in articular cartilage

Immunohistochemical analysis revealed a relative reduction of chondrocytes expressing the NK1-R after 4 weeks of ALN treatment in both Sham and DMM groups in the MFC (Suppl. Figure S6 A) while no differences were detected in the MTP (Suppl. Figure S6B). By trend, a reduction of NK1-R positive chondrocytes after 8 weeks could be detected in all cartilage compartments of the Sham groups. Significant changes were only observed in both the LFC and the LTP cartilage compartments. (Suppl. Figure S6C, D).

A representative IHC staining of NK1-R in cartilage is shown in Supplementary Figure S7.

### ALN treatment effects on the number of MMP-13 and VDIPEN positive cells

Immunohistochemical detection of MMP-13 revealed no significant differences in expression in all compartments neither in the DMM or the Sham group, regardless of ALN administration (Suppl. Figure S8A-D). A representative IHC staining of MMP-13 in cartilage is shown in Supplementary Figure S9.

**Fig. 10:**
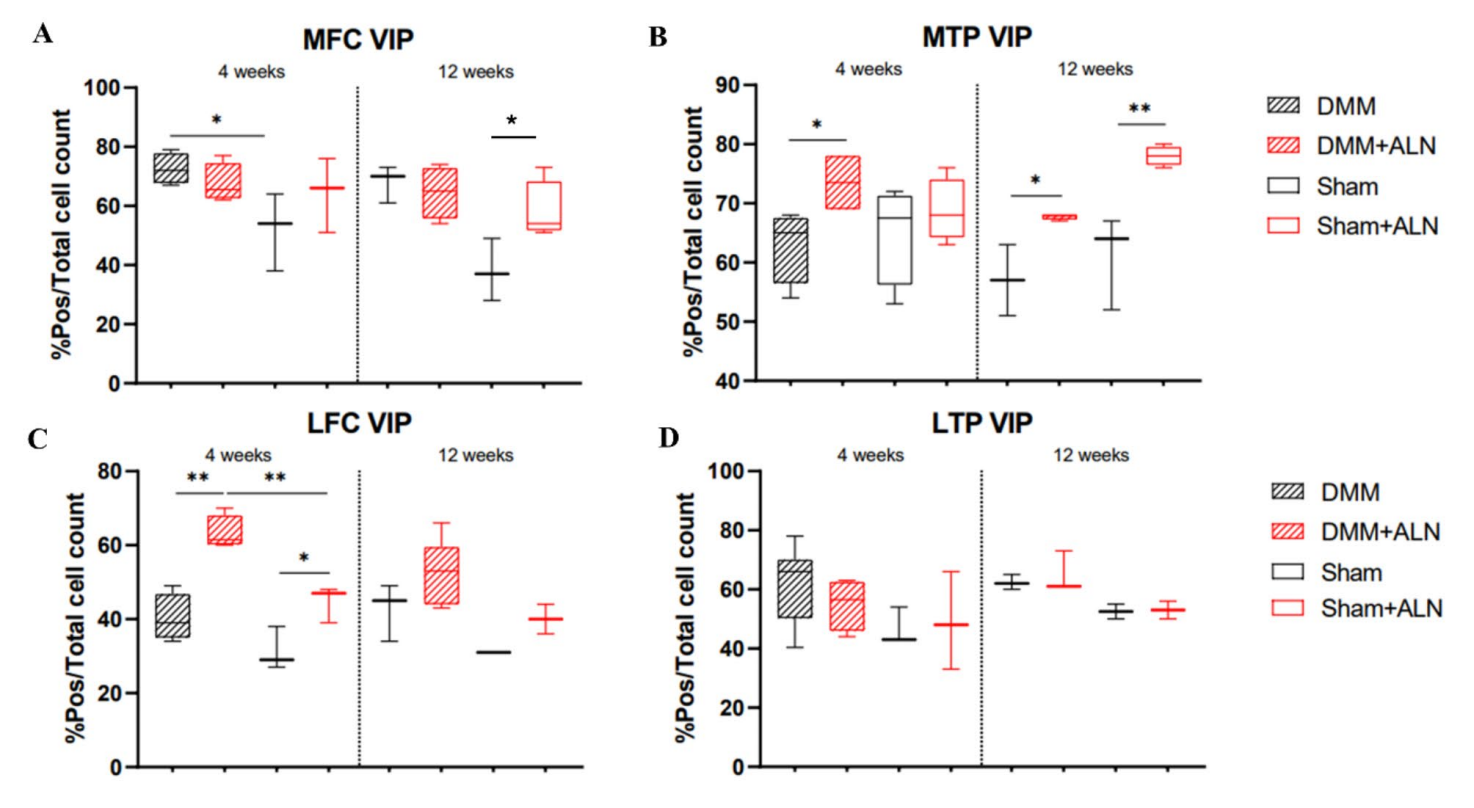
Influence of OA induction and ALN treatment on the number of chondral VDIPEN-positive cells. (**A**-**D**) Number of VDIPEN positive cells were analysed against total chondrocyte count in articular cartilage (in pixel, 1000 px) divided by femur and tibia and medial and lateral compartments of untreated and ALN-treated mice at 4 and 12 weeks after DMM and Sham surgery. Box plots show median and whiskers from min to max. t-test. *p < 0.05, **p < 0.01. *N* = 2–5. MFC = medial femoral condyle; MTP = medial tibia plateau; LFC = lateral femoral condyle; LTP = lateral tibia plateau.

Contrary, under ALN treatment we detected an increased the number of VDIPEN positive cells. VDIPEN is an aggrecan neoepitope formed after cleavage with MMP-13 by cleaving the interglobular domain (IGD) of the core protein of aggrecan. VDIPEN positive cells are significantly increased after ALN treatment in both DMM and Sham groups in all compartments except for the LTP (Fig. 10A-D and suppl. figure S10).

### ALN treatment affects the number of RUNX2 and COMP positive cells and on collagen II expression

Overall, we have not detected significant differences in the ALN treated vs. untreated groups for these molecules. Only in the MTP and MFC, RUNX2 positive cell numbers are significantly increased in the untreated DMM group compared to the Sham group indicating that OA induces an increase in osteoblast number in mechanically loaded knee areas in an early stage of OA. At 12 weeks after DMM significance between those two groups disappeared (Suppl. fig. S11A-D and S12). We did not observe changes in COMP positive cells due to ALN treatment compared with untreated mice except an increase of COMP positive cells in the MTP after 12 weeks between ALN treated DMM and Sham mice (Suppl. fig. S13A-D and S14). Collagen II staining did not reveal obvious differences between the treatment groups at 4 (Suppl. fig. S15A-D) weeks in cartilage (Suppl. fig. S15 A-D).

## Discussion

OA progresses with increasing degenerative changes in joint tissues that ultimately leads to chronic pain and loss of function. To date, no disease-modifying drugs exist that are able to effectively halt the structural decline of bone or cartilage. Often, BPs that are highly efficient in inhibiting increased bone turnover associated with osteoporosis are applied for OA treatment. Early clinical studies suggested effects of BPs in OA, especially in patient-subgroups with BMLs, however follow-up studies were inconclusive^38–40^. Also results from pre-clinical research varied and ranged from no effect to beneficial effects^27–29,41–43^.

### ALN effects on serum markers

In this study, we applied ALN to mice with post-traumatic (PT) OA induced by DMM surgery. A severe side effect of amino-BP treatment is the occurrence of an acute-phase reaction accompanied by the release of pro-inflammatory cytokines such as IL-1β, TNFα and IL-6^44–46^. To our knowledge, no increase of IL-1β, TNFα and IL-6 in serum have been observed so far in OA patients treated with ALN or other BPs. Accordingly, we did not detect altered IL-1β serum concentrations after ALN treatment or OA induction. Presumably, changes in IL-1ß levels might be only detectable in the synovial fluid due to the local but not systemic joint inflammation associated with the DMM model. In line with these observations, we did not detect alterations in serum concentrations of factors related to (the inhibition of) bone degradation (OPG, CTX-I, TRAcP5b), or bone formation (Dkk-1), irrespective of OA induction and/or ALN treatment. However, ADAMTS5, a major enzyme involved in cartilage degradation, was significantly reduced in serum over time after ALN administration in both DMM and Sham mice whereas no effect was observed for the untreated groups during the observation timeline. Notably, neither were ADAMTS5 differences between ALN-treated and untreated groups observed at the same time point indicating an age dependent effect on ADAMTS5 concentration level which is augmented by sustained ALN treatment. Increased levels of ADAMTS5 in serum and synovial fluid are associated with OA, inflammatory changes in joints and mechanical stress^47^. A decrease in ADAMTS5 serum levels therefore suggests a protective effect of ALN treatment against proteoglycan degradation however, independent of OA and rather related to inflammatory processes^48^.

### ALN effects on cartilage degradation

Since reduced ADAMTS5 expression after ALN treatment could potentially have anabolic effects on cartilage metabolism, we then analyzed the structural cartilage and adjacent bone alterations at the knee joint level. Apart from a temporary reduced OARSI score at week 2 in the MTP of ALN treated Sham mice, no differences between ALN treated and untreated mice were observed. Thus, our data suggests no alteration due to repeated high-dose ALN treatment for up to 12 weeks in cartilage degradation in our post-traumatic (PT) OA model. In contrast, a study from Adebayo et al., shows that in two different mouse strains subjected to cyclic compression at the knee joint (5 days a week) cartilage damage might be increased by ALN treatment, especially in C57Bl/6 mice, whereas the ALN-treated FVB mice were protected^27^. Unlike the low-dose treatment with ALN, female C57Bl/6 N mice treated with a high dose of ALN showed a reduced cartilage degeneration early after OA induction by load-induced ACL rupture, but with disease progression, cartilage structure failed similar to the untreated OA mice^28^. Comparison to human OA pathology remains unsatisfactory as treatment start is usually delayed to a point when OA already has been symptomatic, which is usually years after the disease initiation suggesting that the advantage of pre-clinical models is the choice of early treatment starting points. In this regard, Mohan et al. have clearly pointed out that only pre-emptive BP treatment effectively prevented cartilage destruction whereas later onset of treatment had no effect, albeit in an inflammatory OA model (MIA injection model)^41^. The current study included pre-emptive treatment starting from the day of surgery, which may have been too late to observe any beneficial effects, even though a higher dose of ALN was used (1 mg/kg bodyweight vs. 15 µg/kg).

### ALN effects on subchondral bone alterations

After 8 weeks, in depth analysis of bone by ultrahigh-resolution nanoCT analysis revealed OA-related effects and ALN treatment-related effects in the SBP, the epiphyseal and metaphyseal bone. ALN increased Tb.Th. in the tibial epiphysis and metaphysis, whereas BMD was markedly reduced in the epiphyseal region. Positive effects on BV/TV and/or Tb.Th have been reported in a number of studies using ALN in preclinical OA models that fit the anti-resorptive properties of ALN^27,28^. On the other hand, Hayami et al. using a rat ACLT model observed efficient inhibition of bone resorption in early OA by ALN, but furthermore aberrant SB formation (sclerosis) was suppressed^49^. Whether we observed sclerosis or preservation of SB is rather difficult to distinguish. What is clear, however, is that ALN treatment alone induced an osteopetrosis-like phenotype as indicated by increased bone volume fraction and Tb.Th. Notably, this was independent of OA surgery and only observed in the ALN treatment groups.

ALN treatment additionally affected osteophytosis, i.e. the formation of new bone at the joint margins. Osteophytes are a result of chondrogenic differentiation of MSCs of synovial or periosteal origin followed by endochondral ossification and partially by intramembranous bone formation (reviewed in^13^). Furthermore, debate is going on as to whether osteophytes present an adaptation of joint morphology to the occurring biomechanical challenges or are a result of possibly locally altered cell metabolism and biochemical composition of the matrix in the affected joint area.

BPs, especially ALN, displayed high efficacy in inhibiting osteophyte formation or at least in slowing down their maturation^27–29,43^. Here, we found that ALN treatment indeed reduced osteophyte size correlated to the length of the medial condyles of untreated DMM and ALN-DMM mice at 8 weeks. Histology revealed the same trend at the 8 week time point regarding osteophyte size and maturity. However, comparing the 12 weeks time point with the earlier time points (4 and 8 weeks), osteophytes in ALN treated DMM mice were increased in size and maturity whereas osteophytes in untreated DMM-mice did not change size and maturity score indicating a delay in osteophyte growth due to ALN but no prevention.

Remarkably, BMD was decreased in the epiphyseal bone, in osteophytes as well as in the meniscal ossicles in ALN treated mice. In general, ALN actions are associated with restoration or increase of BMD making it the therapy of choice in conditions with increased bone turnover, e.g. osteoporosis (reviewed in^50^, Paget disease of bone^51^, and tumor-related complications^52^. Furthermore, ALN treatment after arthroplasty positively affected BMD of the proximal femur and distal tibia and suggested positive effects on implant holding^53^. The impact on BMD might be site specific as lumbar spine BMD increased whereas hip BMD remained unaffected by ALN in osteoporotic patients after gastrectomy^54^ concordant with unchanged BMD in the metaphyseal bone in our study. In general, osteophytes and meniscal ossicles represent new bone deposition by endochondral ossification and heterotopic ossification, respectively, and the epiphysis is associated with altered bone turnover in OA compared to less-affected compartments such as the metaphysis. Therefore, alterations in BMD might be more pronounced in anatomical regions of new bone formation. Moreover, there are certain indications that BP might reduce BMD in the first place. Kendler et al. described in their randomized, open-label, two-year crossover study that a proportion of patients, transitioning from denosumab to ALN treatment, experienced a reduction in BMD of lumbar spine, hip or femoral neck, though losses were moderate and most patients retained BMD above baseline^55^. The moderate loss is concordant with our results that describe a BMD reduction around 3–4% in the epiphysis and around 2% in the meniscal ossicles. Kendler and colleagues attributed the site-specific effects to differences in proportion of cortical vs. trabecular bone and mechanical parameters^55^. Iwata and colleagues observed a reduced mineral apposition and bone formation rate after risedronate or ALN application in the rat tibia^56^. However, in vitro studies suggest that mineralization might be affected by BPs in a concentration- and time-dependent manner. Especially high doses of BP might interfere with mineralization by affecting osteoblast-mediated mineral apposition. We observed a BMD reduction after 8 weeks of treatment with high-dose ALN, indicating a negative impact on osteoblast induced mineralization. Gronowicz et al. provided evidence that ALN might regulate aberrant osteoblast properties^57^. In their study, ALN could normalize hypermineralization of sclerotic osteoblasts derived from otosclerosis patients to levels of normal human osteoblasts. The dose and duration of the treatment, as well as cellular predisposition of osteoblasts might therefore determine the reactivity to BP treatment, making it essential to carefully evaluate the dose and treatment regimen, in particular for small animal OA models.

Determining the number of osteoclasts revealed an increase initially observed after 4 weeks of ALN therapy. In the further course, the number of osteoclasts stagnated and at a later point in time there was a decrease in osteoclasts. Our intermittent ALN application schedule (2x per week) might stimulate differentiation of precursor cells, i.e. macrophages to osteoclasts as a rescue response to reduction of precursor differentiation directly after ALN application. This would be according to the observation of the group of Forwood who observed a significant interaction between the effects of time and treatment type on the number of osteoclasts per µm^2^ unit BMU area and length. The number of osteoclasts per µm^2^ unit BMU area and length was significantly greater in ALN cessation groups at 2 weeks after fracture induction in a rat ulna stress fraction model compared to the continuous ALN application group^58^.

In general, the alterations in bone structure caused by ALN-treatment might partly indicate a beneficial mechanism. Strengthening of SB will support and protect the overlying articular cartilage from instability-induced structural deterioration. Targeting and interfering with bone remodeling for a continued time span might in turn lead into detrimental effects as a reduced bone turnover can potentially result in improperly mineralized brittle bone structures prone to micro-damage.

### ALN effects on total cell count in cartilage

When assessing the total chondrocyte count, we found a significant difference between the treated and non-treated group in the LTP after 8 weeks. ALN treatment induced a significant increase in cell count in both the DMM and Sham groups. There were no differences in the other compartments except temporarily at 4 weeks in the MTP. An effect of high-dose alendronate treatment on the number of chondrocytes is currently not described in the literature. Ultimately, our study only showed an increase in chondrocyte count in one compartment. Further in vitro studies would be necessary to obtain molecular data on the proliferation of chondrocytes when treated with ALN in order to demonstrate whether the change in cell count is physiologically relevant.

### ALN effects on NK1-R and Runx2 expression in chondrocytes and cleavage of aggrecan

The proportion of NK1-R-positive chondrocytes was compared in DMM and Sham mice 4 and 8 weeks postoperatively under ALN treatment. We found that ALN treatment of sham mice reduced the relative number of NK1-R-positive chondrocytes in all cartilage compartments except in the MTP. Some studies have already examined the role of NK1-R in the development and progression of degenerative joint diseases. Adult patients with rheumatoid arthritis and patients with OA had increased expression of NK1-R and increased SP levels in synovial fibroblasts, indicating an increased catabolic effect in cartilage in OA^59^. In contrast, a study by Muschter et al. (2020), using a SP-deficient DMM OA model, revealed that the lack of SP exerts a catabolic effect on cartilage tissue^32^. In 2012, a study by Opolka et al. showed, that stimulation of neonatal rib chondrocytes with SP leads to an increased proliferation rate of these chondrocytes isolated from newborn mouse rib cages in cell culture^60^. Comparing these studies with our actual data regarding the influence of ALN on the expression of NK1-R, we observed that ALN tended to inhibit the expression of NK1-R. There is currently no information available in the literature about the effect of ALN treatment on the expression of the NK1-R in peripheral tissues. However, in the future, more detailed knowledge about the role of tachykinin receptors and the influence of drug therapies on chondrocyte metabolism could represent a possible therapeutic approach regarding OA pain or symptomatic inflammation.

Additionally, we have analysed protein expression of catabolic markers as MMP-13 and VDIPEN in cartilage. VDIPEN is an aggrecan neoepitope (Asn341-Phe342) after cleavage with MMP-13 by cleaving the interglobular domain (IGD) of the core protein of aggrecan^21^. We detected an increase of VDIPEN positive cells after ALN treatment however, independent of OA. This observation indicated a higher activity of MMP-13 under ALN treatment as we did not detect differences in numbers of MMP-13 positive cells suggesting no changes in protein synthesis but increased activation of MMP-13 by ALN. Anabolic cartilage markers as COMP and collagen II remained mostly unchanged in all groups. Increase in RUNX2 positive cells in the untreated DMM groups at an early OA time point in the medial compartments indicated that OA induces an increase in hypertrophic chondrocyte numbers expressing the osteoblast marker RUNX2 in mechanically loaded knee areas in an early stage. 12 weeks after DMM significance between those two groups disappeared. A limitation of this basic research study is the lack of pain related analysis as ALN treatment in the case of osteoporosis also confers pain relief. However, the intention of this study was to analyse the underlying molecular mechanisms of ALN treatment in a surgical preclinical OA mouse model and thus clinical aspects like pain sensation would have been out of scope.

## Conclusion

In this study, we analyzed the effects of high-dose ALN application on the progression of OA in the mouse DMM-OA model on the cartilage and bone level. ALN application induced an increase in SBP thickness accompanied with an increased bone volume fraction and trabecular thickness and number in the epiphysis and medial subchondral tibia. In addition, ALN was able to reduce the formation of osteophytes at early time points but ALN treatment also induced meniscal ossicle formation after OA induction. Lastly, no protective effect on progressive cartilage destruction in the DMM groups was observed after ALN administration.

With regard to the proportion of NK1-R-positive chondrocytes and the total cell count as possible indicators of inflammation-related changes in metabolism, it can be summarized that high-dose ALN administration reduces the expression of NK1-R, especially in the cartilage of Sham mice; the total number of chondrocytes in both surgery groups was significantly increased only in the LTP after ALN administration. However, a resulting slower progression of cartilage matrix destruction was not observed according to the OARSI score possibly due to an increase in aggrecan cleavage demonstrated by immunostaining of its neoepitope VDIPEN together with no (COMP and collagen II) or only temporary effects (RUNX2) on anabolic cartilage and bone markers. In addition, a reduction in serum ADAMTS5 level over time was observed after ALN administration, without a correlation to articular cartilage degradation.

Overall, ALN cannot be recommended as potential treatment strategy for general OA therapy in later stages due to mostly detrimental effects on bone and partly also cartilage structures.

However, it might be considered as a potential treatment strategy in a subgroup of OA patients with high bone turnover in an early-stage of OA, but this requires in depth further investigation of the treatment schedule and dosing in pre-clinical animal OA models.

## Electronic supplementary material

Below is the link to the electronic supplementary material.

Supplementary Material 1

Supplementary Material 2 I have done some minor corrections in the supplementary material 2, however, I could not save it, so I have uploaded it again.

## Data Availability

Data is provided within the manuscript or supplementary information files.

## Abbreviations

ADAMTS-5: Disintegrin- and metalloproteinase with thrombospondin motifs
ALN: Alendronate
BMD: Bone mineral density
BP: Bisphosphonate
BS: Bone surface
BV: Bone volume
CC: Calcified cartilage
CGRP: Calcitonin-gene-related-peptide
COMP: Cartilage oligomeric matrix protein
CTX-I: Cross-Linked C-Telopeptide-I
Dkk-1: Dickkopf-1
DMM: Destabilized medial meniscus
IL (-1β, -6, -10): Interleukins
LFC: Lateral femur condyles
LTP: Lateral tibia plateau
MMP: Matrix metalloproteinase
MFC: Medial femur condyles
MTP: Medial tibia plateau
NK1-R: Neurokinin 1-receptor
OA: Osteoarthritis
OPG: Osteoprotegerin
PT: Post-traumatic
px: Pixel
RANKL: Receptor activator of NF-κB-Ligand
ROI: Region of interest
TNFα: Tumor necrosis factor alpha
TRAcP5b: Tartrate-resistant acid phosphatase 5b
TV: Total volume
VOI: Volume of interest
