# Supplementary material for: Differential effects of alendronate on chondrocytes, cartilage matrix and subchondral bone structure in surgically induced osteoarthritis in mice
Source: Sci Rep. 2024 Oct 23;14:25026. doi: 10.1038/s41598-024-75758-7 (PMC11500094; doi:10.1038/s41598-024-75758-7)
Supplement: Supplementary file 2 — Supplementary Material 2 I have done some minor corrections in the supplementary material 2, however, I could not save it, so I have uploaded it again. [file 41598_2024_75758_MOESM2_ESM.docx]

**Supplementary figures S1-15**


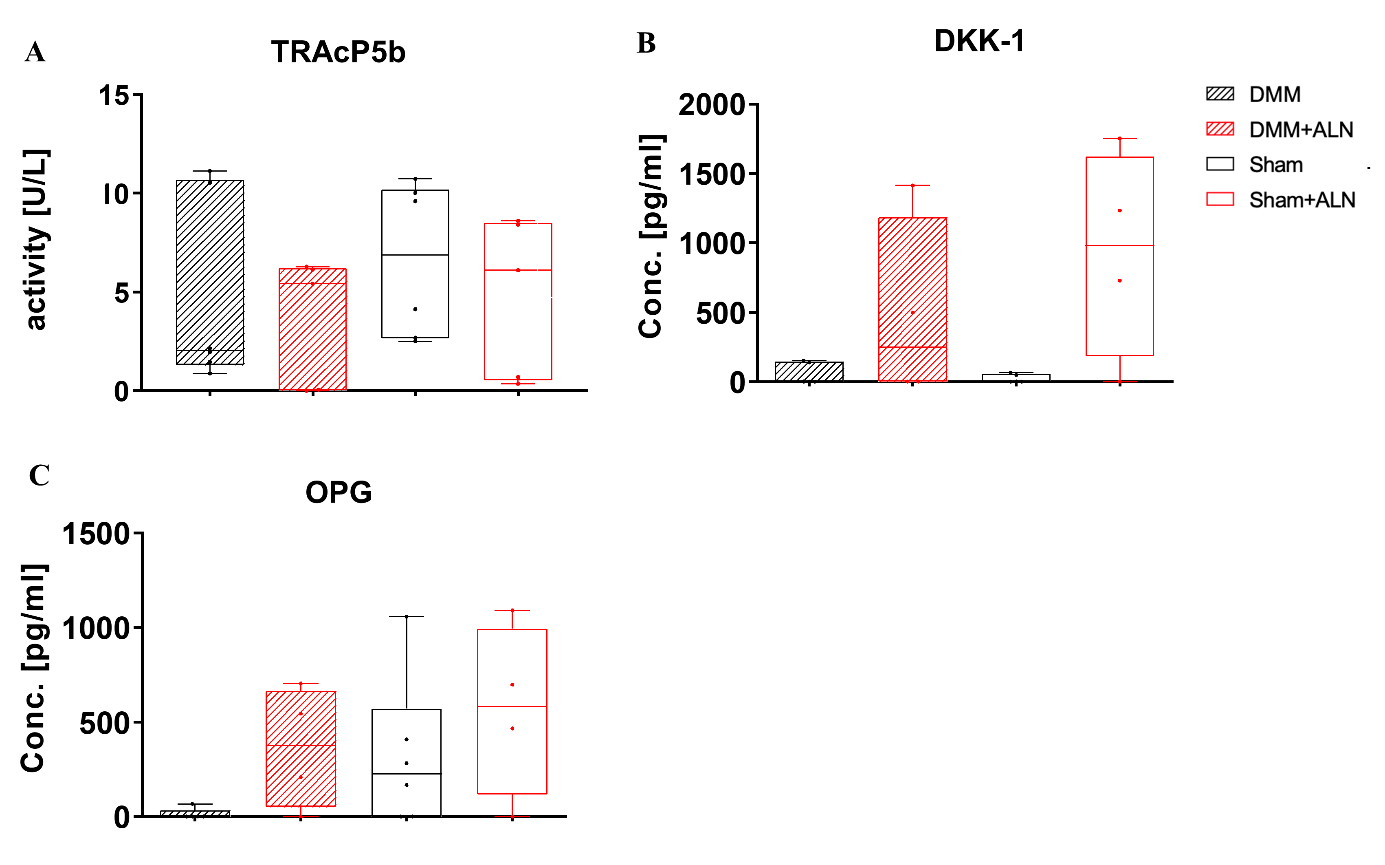


**Supplementary Figure S1: Serum marker analysis for osteoclast and osteoblast modulation in untreated and ALN-treated mice in late stage OA.**

Serum concentration of osteoclast activity marker tartrate-resistant acid phosphatase 5b (TRAcP5b; A), osteoblast activity inhibitor dickkopf-1 (Dkk-1; B) and decoy receptor for Rankl, osteoprotegerin (OPG; C) were analyzed by ELISA in untreated and ALN-treated mice 12 weeks after DMM or Sham surgery. Box plots show median and whiskers from min to max. One-way Anova, N=4-6.


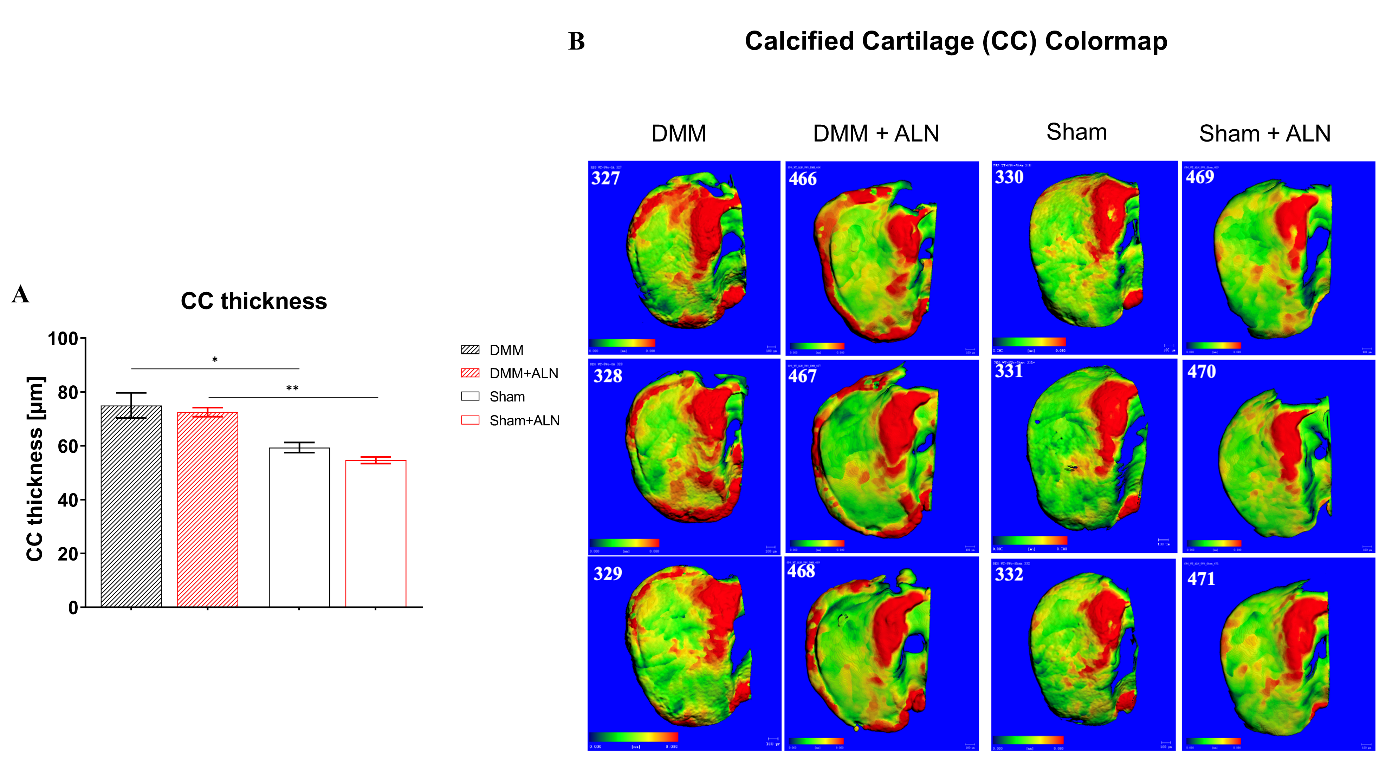


**Supplementary Figure S2: NanoCT analysis of the medial tibial calcified cartilage thickness of untreated and ALN-treated mice after DMM and Sham surgery.**

A) Thickness of the calcified cartilage (CC) layer of the medial tibial plateau was extracted by differential grey value analysis using nanoCT analysis. CC thickness was compared in untreated and ALN-treated mice 8 weeks after DMM or Sham surgery. Bars with median and 95% confidence interval. One-way ANOVA followed by Bonferroni post-hoc test. *p<0.05, **p<0.01. N=3.

B) The colormap shows, that ALN treatment did not affect the mean CC thickness. The color maps indicate that the CC overlying the osteophyte rim appears to be thicker in the ALN-DMM group than in the untreated DMM group (more red spots over the rim). This indicates that the osteophytes in the ALN-treated groups are less mineralized than in the untreated groups.


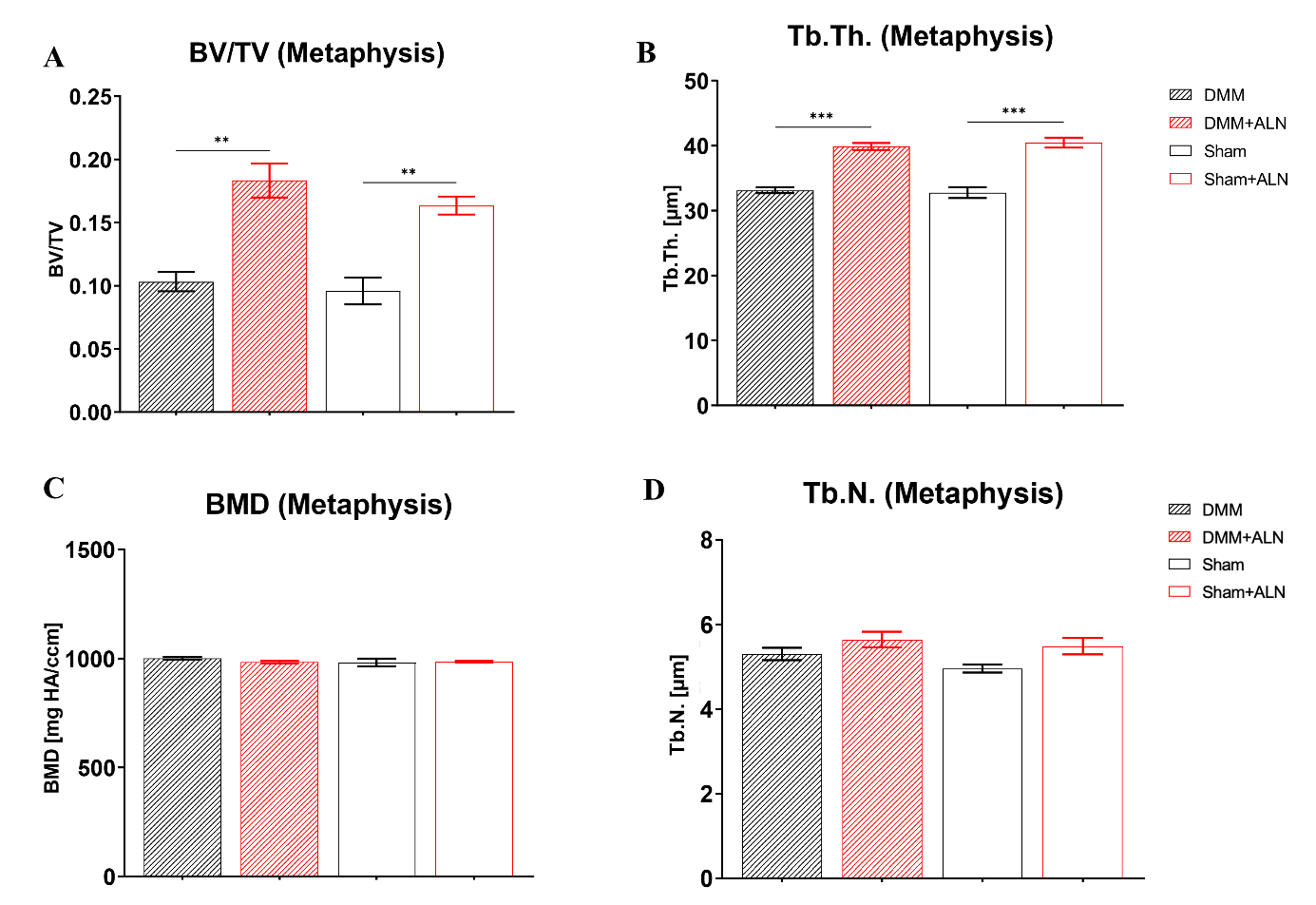


**Supplementary Figure S3: NanoCT analysis of the metaphyseal bone of untreated and ALN-treated mice after DMM and Sham surgery.**

NanoCT analysis of the metaphyseal trabecular tibial bone of untreated and ALN-treated mice 8 week after DMM or Sham surgery including bone volume density (BV/TV; A), trabecular thickness (Tb.Th.; B), bone mineral density (BMD; C) and trabecular number (Tb.N.; D). Bars with median and 95% confidence interval. One-way ANOVA followed by Bonferroni post-hoc test. **p<0.01, ***p<0.001. N=3.


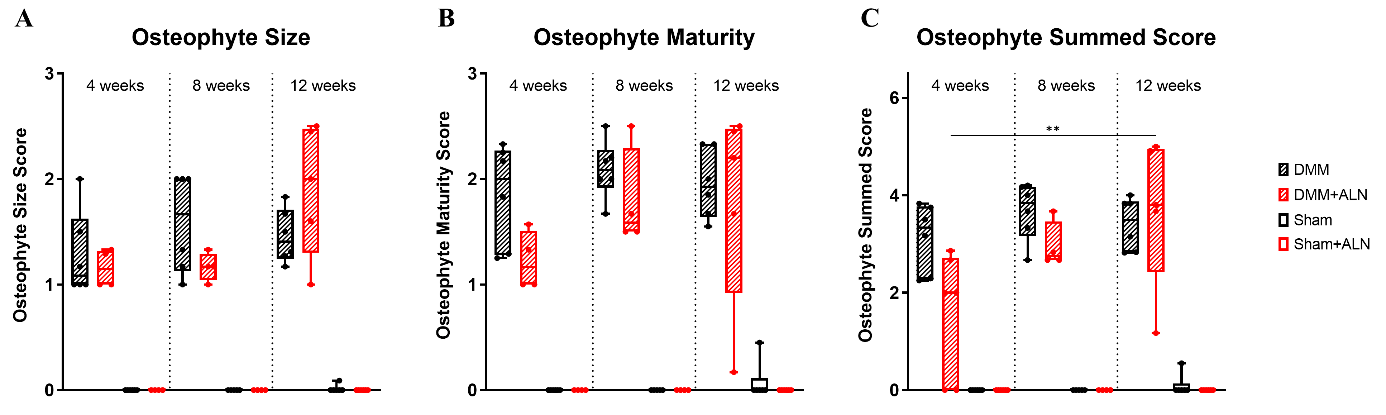


**Supplementary Figure S4: Characterization of osteophytosis after DMM surgery in untreated and ALN treated mice**

Osteophyte grading of size (A), maturity (B) and the summed score (C) in untreated and ALN treated mice at 4, 8 and 12 weeks after DMM and Sham surgery. One way ANOVA followed by Bonferroni post-hoc test. *p < 0.05. N = 4-6.


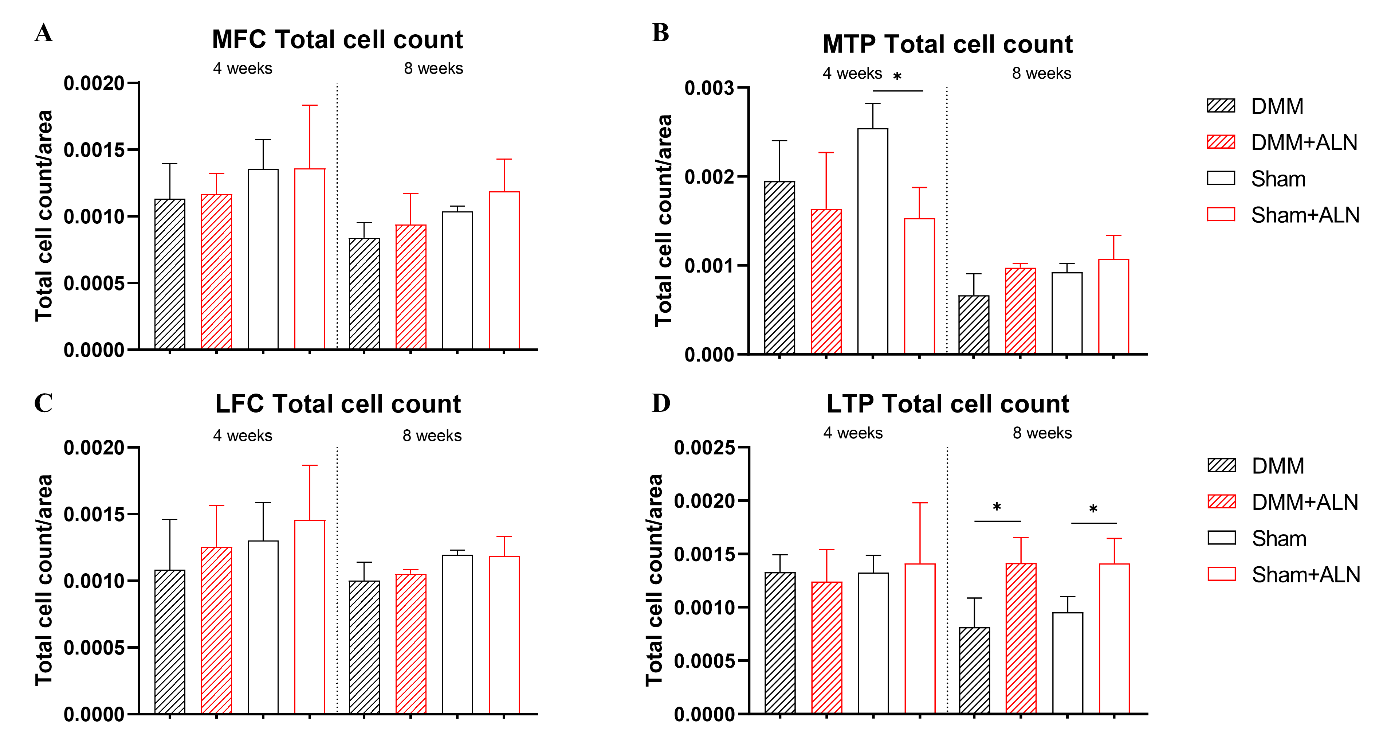


**Supplementary Figure S5: Influence of OA induction and ALN treatment on the total chondrocyte count.**

A-D) Total chondrocyte count in superficial articular cartilage divided by femur and tibia and medial and lateral compartments of untreated and ALN-treated mice at 4 and 8 weeks after DMM and Sham surgery. Box plots show median and whiskers from min to max. t-test. *p<0.05. N=3.

MFC= medial femoral condyle; MTP= medial tibia plateau; LFC= lateral femoral condyle; LTP= lateral tibia plateau


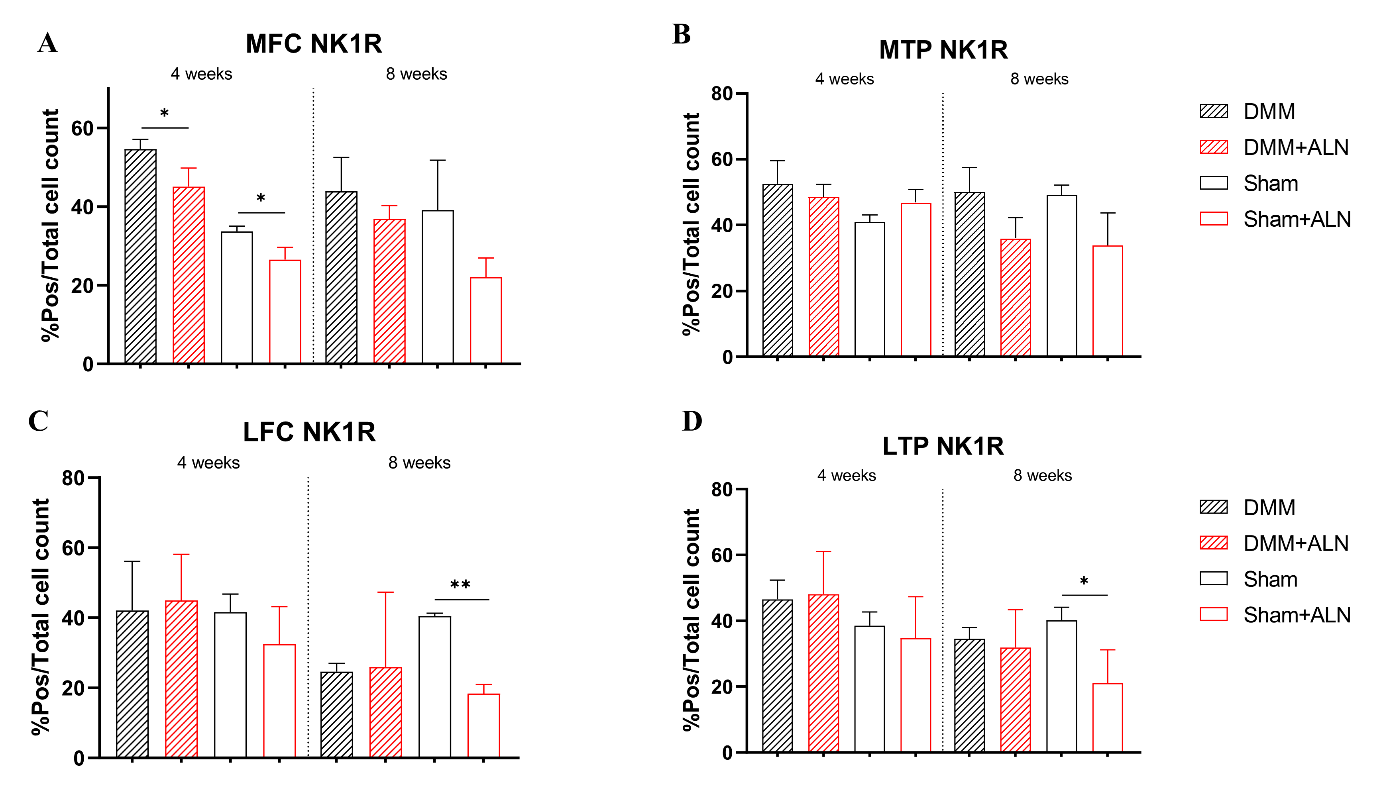


**Supplementary Figure S6: Influence of OA induction and ALN treatment on the number of NK1-R positive chondrocytes.**

A-D) Number of NK1-R positive chondrocytes against total chondrocyte count in superficial articular cartilage (in pixel, 1000 px) divided by femur and tibia and medial and lateral compartments of untreated and ALN-treated mice at 4 and 8 weeks after DMM and Sham surgery. Box plots show median and whiskers from min to max. t-test. *p<0.05, **p<0.01. N=3

MFC= medial femoral condyle; MTP= medial tibia plateau; LFC= lateral femoral condyle; LTP= lateral tibia plateau


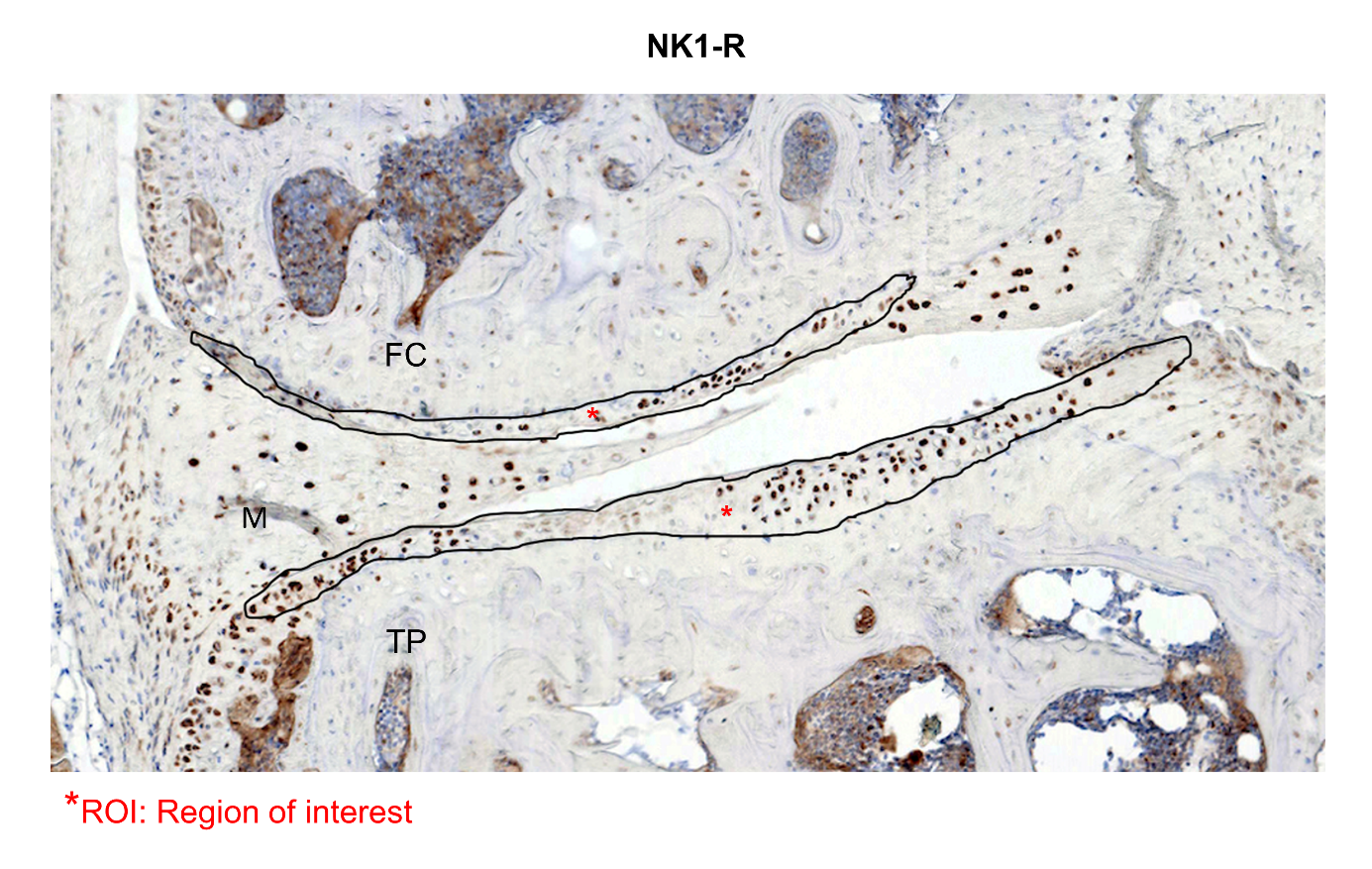


**Supplementary Figure S7: Immunohistochemical staining of NK1-R in chondrocytes.**

Representative image of immunohistochemical staining of the NK1-R; the counted area is outlined in black. FC= femoral condyle, TP= tibia plateau, M= meniscus.

10x magnification was used for counting and photographing.


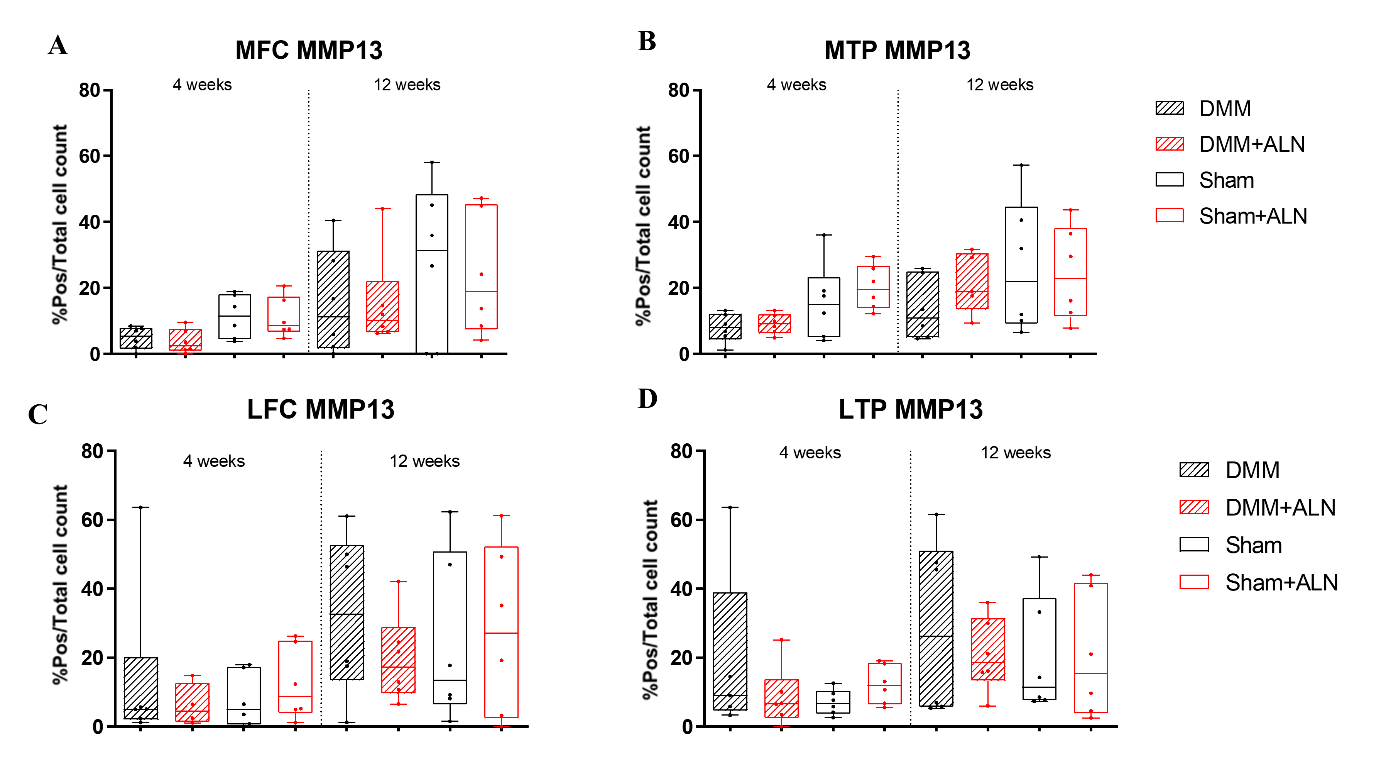


**Supplementary Figure S8: Influence of OA induction and ALN treatment on the number of MMP13 positive chondrocytes.**

A-D) Number of MMP13 positive chondrocytes counted against total chondrocyte numbers in superficial articular cartilage (in pixel, 1000 px) divided by femur and tibia and medial and lateral compartments of untreated and ALN-treated mice at 4 and 12 weeks after DMM and Sham surgery. Box plots show median and whiskers from min to max. t-test. One-way Anova, N=6.

MFC= medial femoral condyle; MTP= medial tibia plateau; LFC= lateral femoral condyle; LTP= lateral tibia plateau


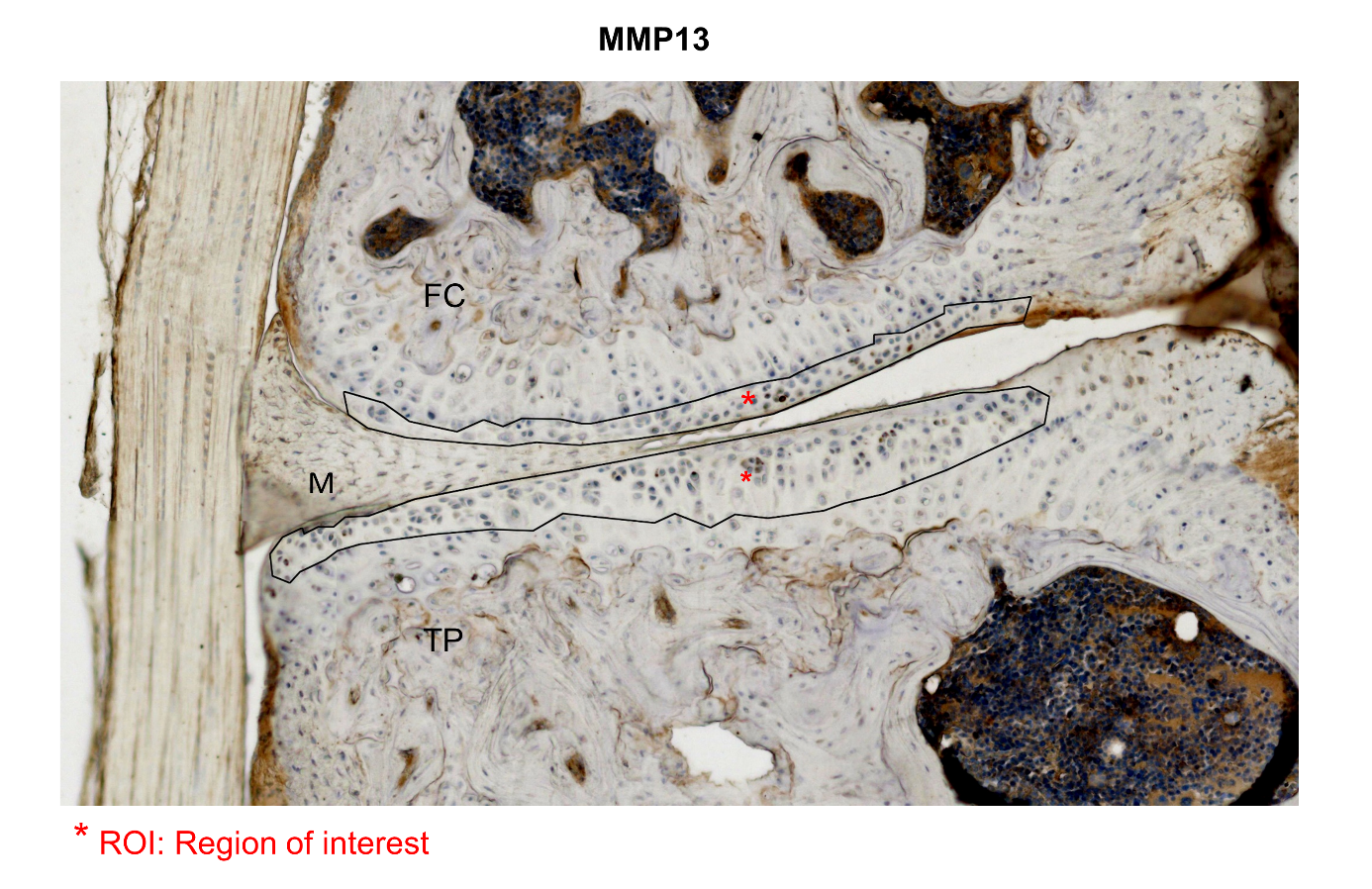


**Supplementary Figure S9: Immunohistochemical staining of MMP13 in chondrocytes.**

Representative image of immunohistochemical staining of MMP13; the counted area is outlined in black. FC= femoral condyle, TP= tibia plateau, M= meniscus.

10x magnification was used for counting and photographing.


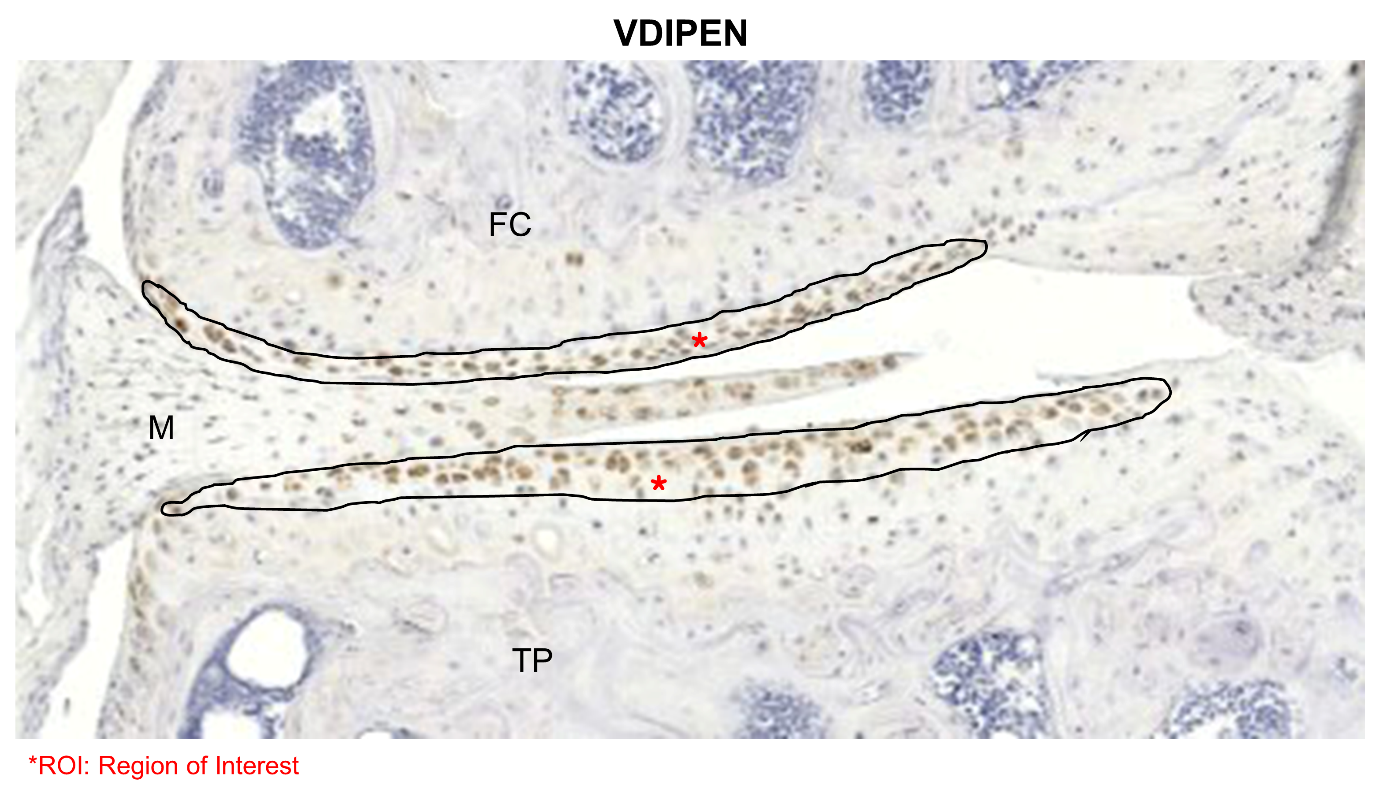


**Supplementary Figure S10: Immunohistochemical staining of VDIPEN in chondrocytes**.

Representative image of immunohistochemical staining of aggrecan neoepitope VDIPEN; the counted area is outlined in black; FC=: femoral condyle, TP= tibia plateau, M= meniscus. 10x magnification was used for counting and photographing.


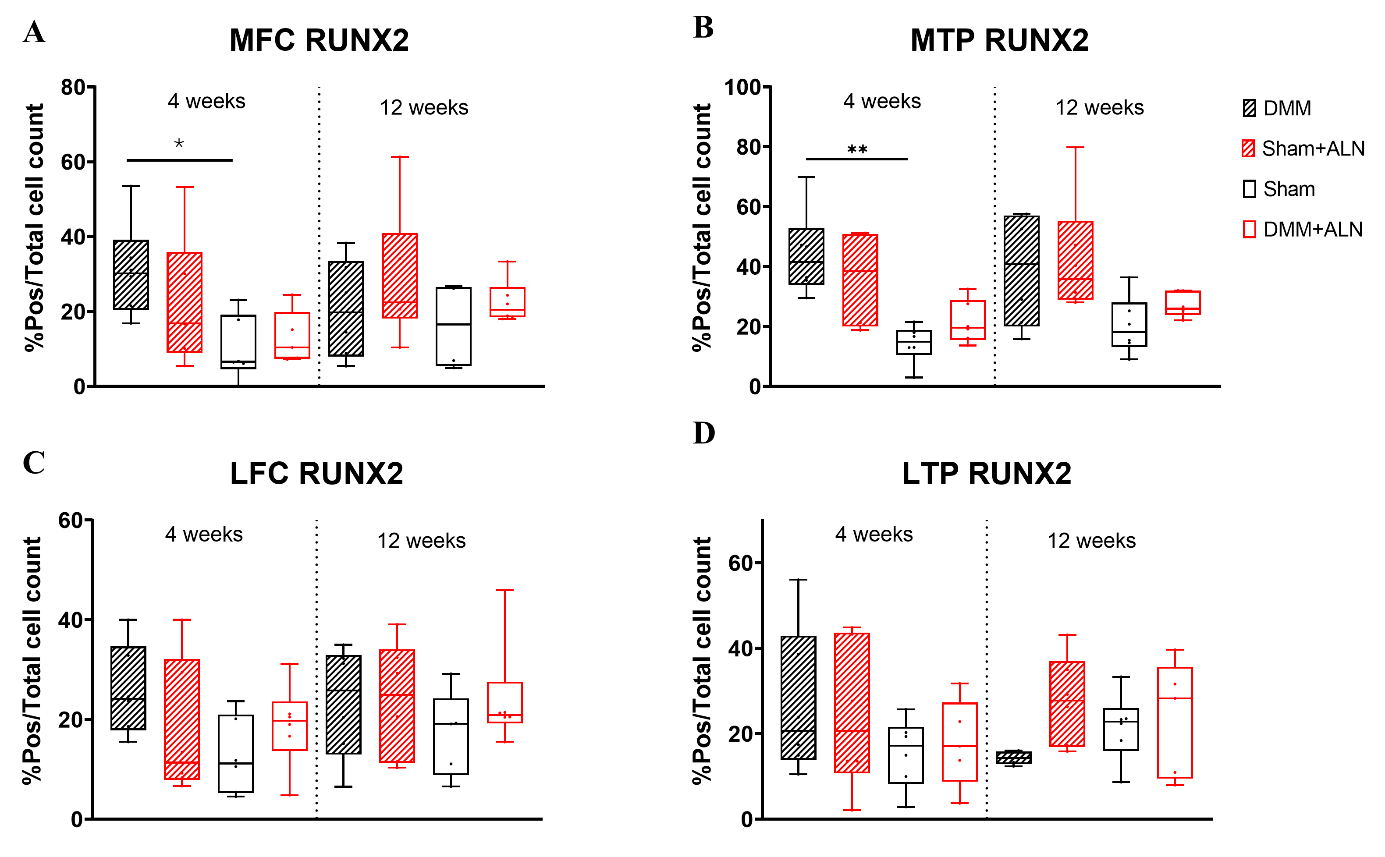


**Supplementary Figure S11: Influence of OA induction and ALN treatment on the number of chondral RUNX2 positive cells.**

A-D) Number of RUNX2 positive cells counted in relation to total chondrocyte count in articular cartilage (in pixel, 1000 px) divided by femur and tibia and medial and lateral compartments of untreated and ALN-treated mice at 4 and 12 weeks after DMM and Sham surgery. Box plots show median and whiskers from min to max. t-test. *p<0.05, **p<0.01. N=4-6. MFC= medial femoral condyle; MTP= medial tibia plateau; LFC= lateral femoral condyle; LTP= lateral tibia plateau


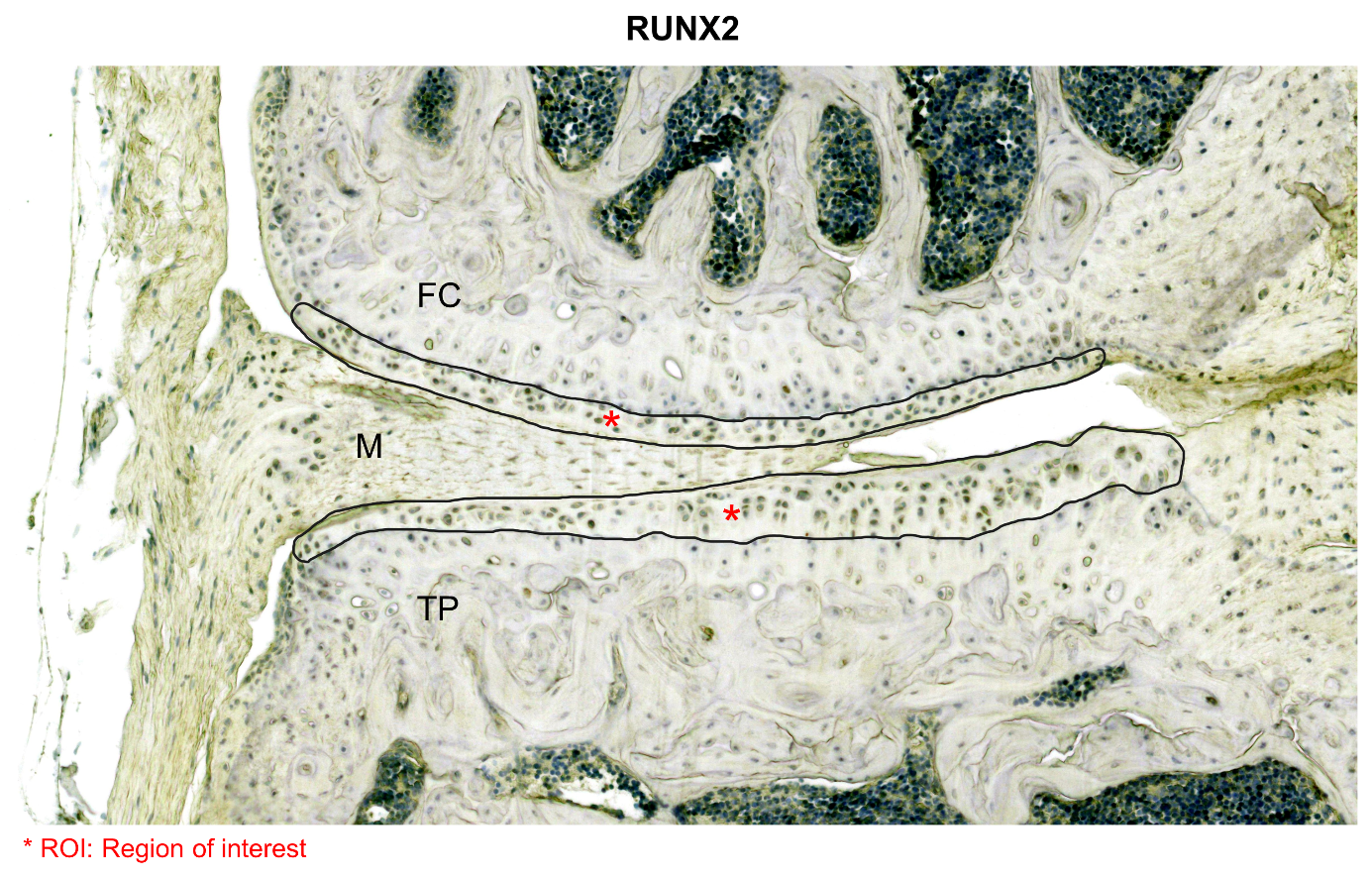


**Supplementary figure S12: Immunohistochemical staining of RUNX2 in cartilage.**

Representative image of the medial knee of an ALN-treated mouse 12 weeks after Sham surgery stained for RUNX2. The counted area is outlined in black, FC=femoral condyle, TP=tibia plateau, M=meniscus. 10x magnification for counting and photographing.


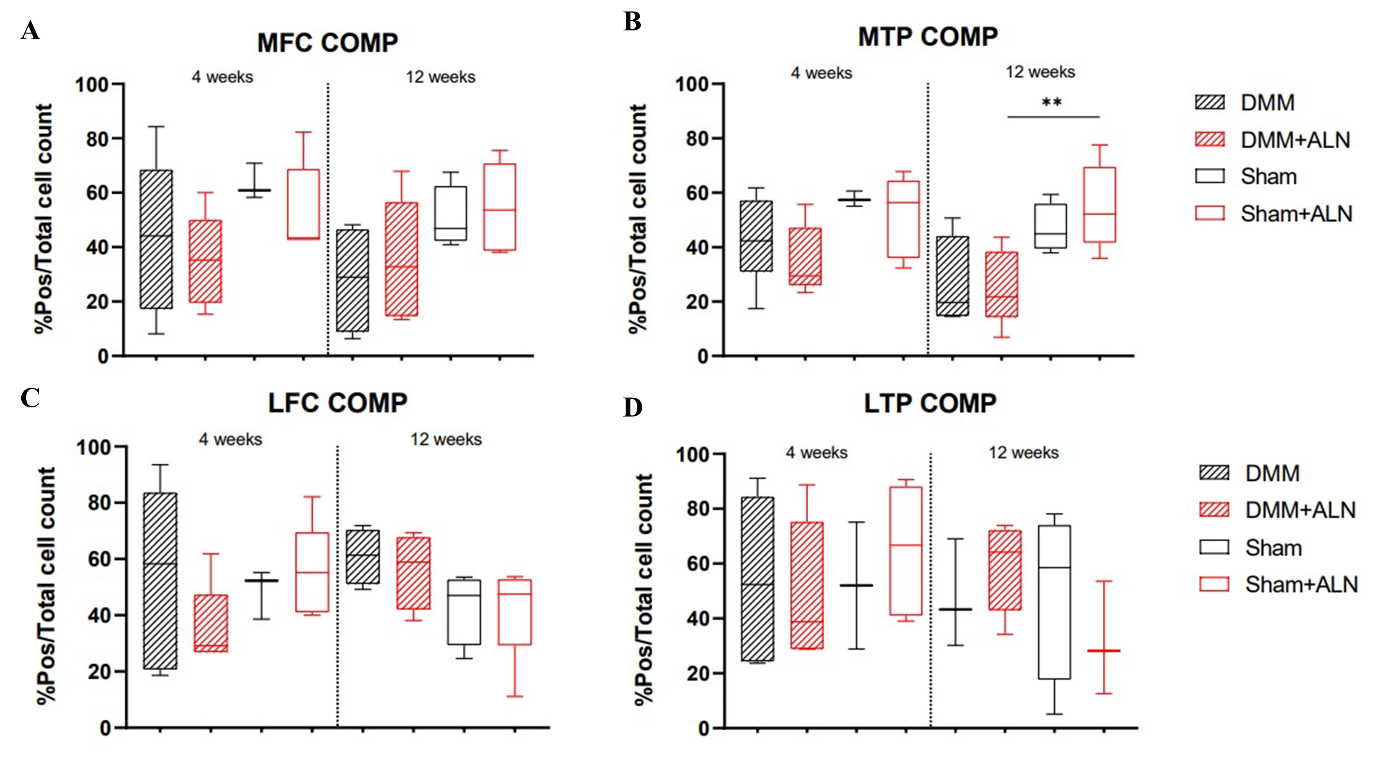


**Supplementary figure S13: Influence of OA induction and ALN treatment on the number of COMP-positive chondrocytes.**

A-D) Number of COMP positive cells counted in relation to the total chondrocyte count in articular cartilage (in pixel, 1000 px) divided by femur and tibia and medial and lateral compartments of untreated and ALN-treated mice at 4 and 12 weeks after DMM and Sham surgery. Box plots show median and whiskers from min to max. One-way ANOVA followed by Kruskal-Wallis-test. *p<0.05, **p<0.01, ***p<0.001, ****p<0.000. N=2-5.

MFC= medial femoral condyle; MTP= medial tibia plateau; LFC= lateral femoral condyle; LTP= lateral tibia plateau


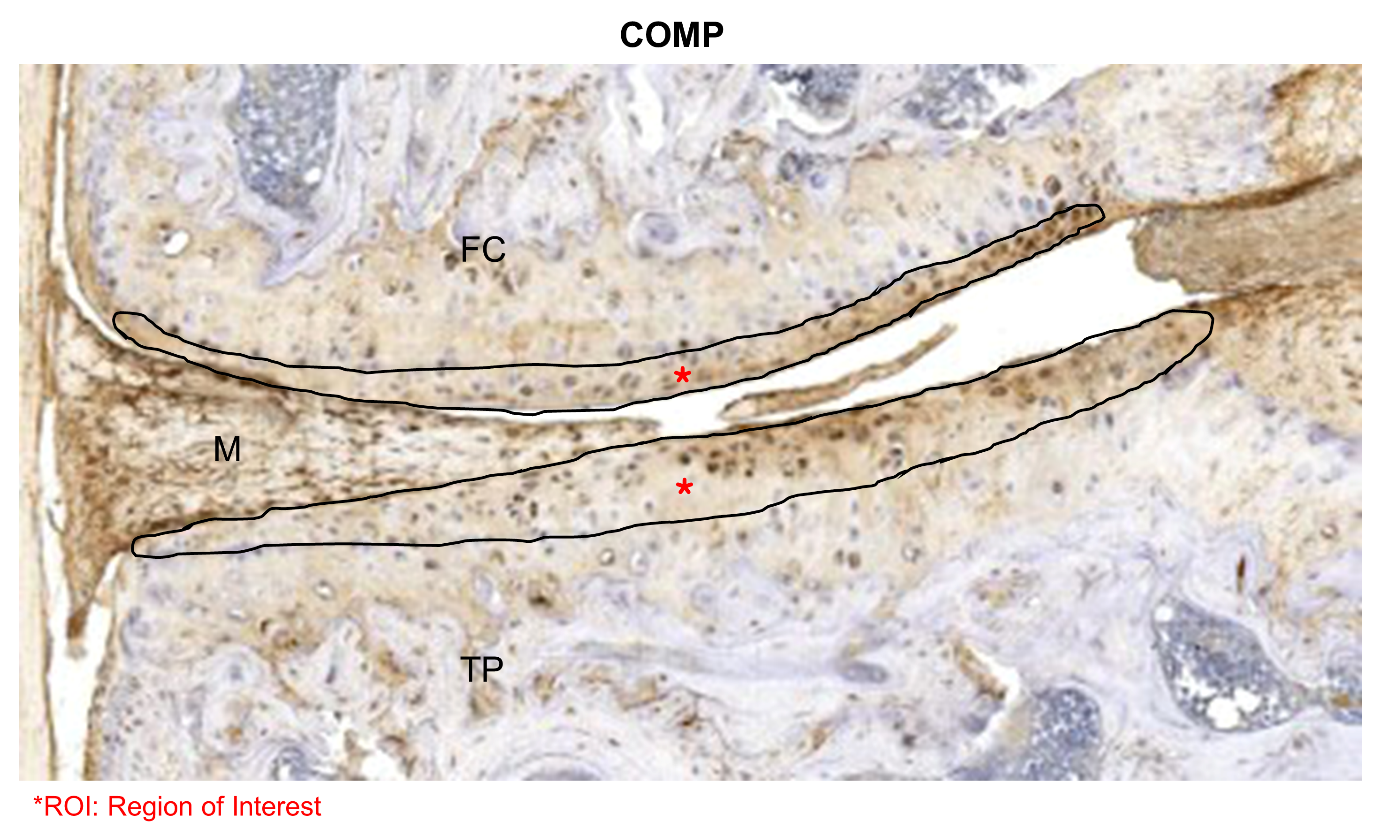


**Supplementary Figure S14: Immunohistochemical staining of COMP in chondrocytes.**

Representative image of the medial knee of an ALN-treated mouse 12 weeks after Sham surgery stained for COMP. The counted area is outlined in black, FC=femoral condyle, TP=tibia plateau, M=meniscus. 10x magnification for counting and photographing.


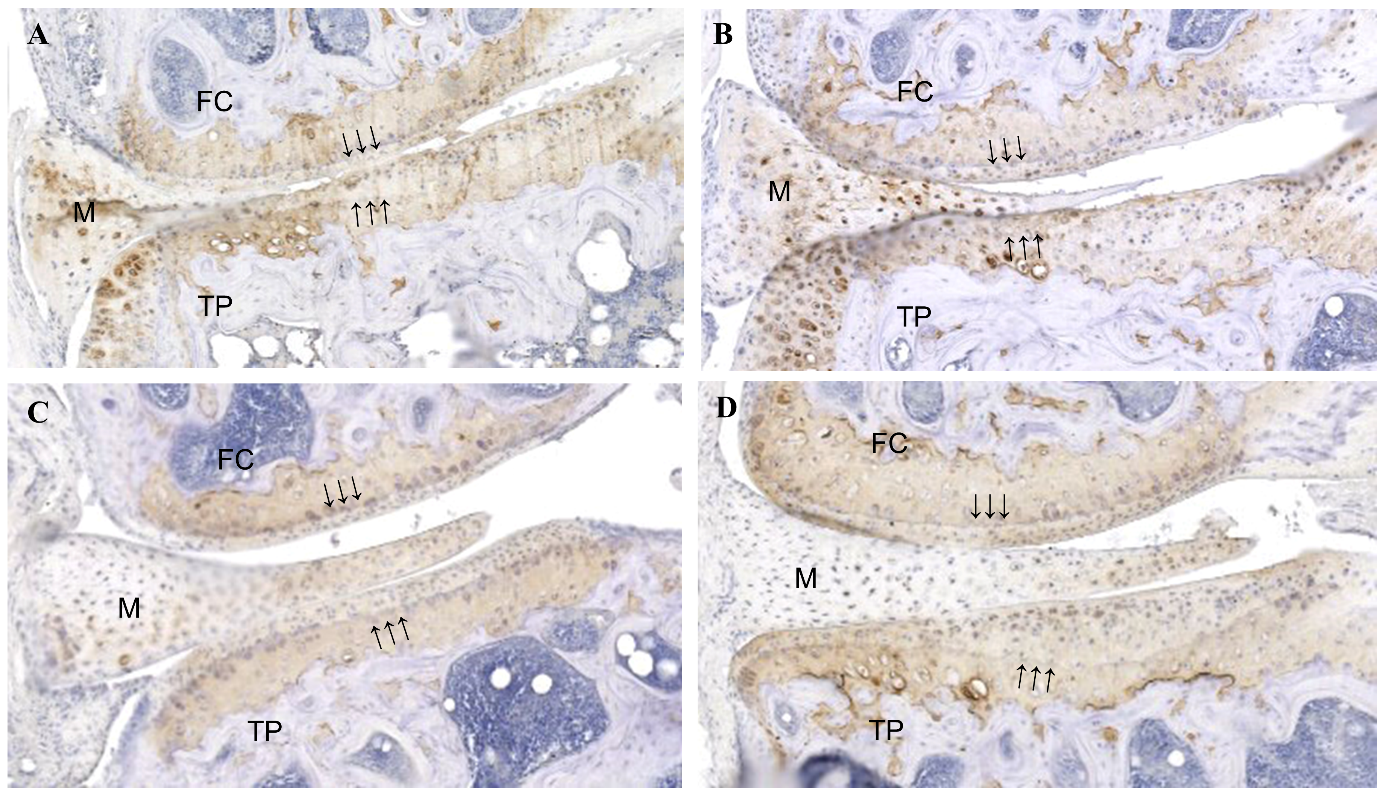


**Supplementary Figure S15: Immunohistochemical staining of collagen II in cartilage.**

Representative images of the medial knee of ALN-treated and untreated mouseat 4 weeks after DMM and Sham surgery stained for collagen II.

A) DMM; B) DMM + ALN; C) Sham; D) Sham + ALN.

The arrows show the tidemark. The intensity of the staining of the matrix is assessed qualitatively; FC=: femoral condyle, TP= tibia plateau, M= meniscus. The arrows show the cartilage-bone boundary FC=femoral condyle, TP=tibia plateau, M=meniscus.

10x magnification is used for photographing.
